# A Comprehensive Review on LncRNAs/miRNAs‐DNMT1 Axis in Human Cancer: Mechanistic and Clinical Application

**DOI:** 10.1111/jcmm.70604

**Published:** 2025-05-19

**Authors:** Seyed Mohsen Aghaei‐Zarch, Ali Esmaeili, Saeid Bagheri‐Mohammadi

**Affiliations:** ^1^ Department of Medical Genetics, School of Medicine Shahid Beheshti University of Medical Sciences Tehran Iran; ^2^ Student Research Committee, Department of Tissue Engineering and Applied Cell Sciences, School of Advanced Technologies in Medicine Shahid Beheshti University of Medical Sciences Tehran Iran; ^3^ Department of Paramedicine, Amol School of Paramedical Sciences Mazandaran University of Medical Sciences Sari Iran; ^4^ Immunogenetic Research Center Mazandaran University of Medical Sciences Sari Iran

**Keywords:** cancer, *DNMT1*, long non‐coding RNAs, microRNAs, non‐coding RNAs, therapy

## Abstract

Cancer constitutes a significant public health concern, and addressing the challenge of cancer holds paramount importance and requires immediate attention. Epigenetic alterations, encompassing DNA methylation, have emerged as pivotal contributors to the development of diverse cancer types. These modifications exert their influence by modulating chromatin structure, gene expression patterns and other nuclear processes, thereby influencing cancer pathogenesis. Over the last two decades, an increasing body of evidence has established the involvement of DNA methyltransferase 1 (*DNMT1*) in various aspects of cancer development, including tumorigenesis, aggressiveness and treatment response. Furthermore, non‐coding RNAs (ncRNAs), such as microRNAs (miRNAs) and long non‐coding RNAs (lncRNAs), are increasingly recognised as significant modulators in diverse biological processes, encompassing metastasis, apoptosis, cell proliferation and differentiation. Several recent studies have elucidated the intricate relationship between epigenetic machinery, specifically *DNMT1*, and the expression of ncRNAs in the context of cancer. In this review, we provide a comprehensive overview of the interaction between *DNMT1* and ncRNAs in cancer pathogenesis. Furthermore, we discuss the important role of the ncRNAs‐*DNMT1* axis in cancer stem cells and cancer therapy resistance as critical issues in cancer therapy. Finally, we demonstrate that herbal medicine and synthetic RNA molecules regulate *DNMT1* activity and hold great promise in cancer treatment.

**Abbreviations**
5‐AzadC: 5‐Aza‐2′‐deoxycytidine
ALCL: anaplastic large‐cell lymphoma
ALL: acute lymphoblastic leukaemia
AML: acute myeloid leukaemia
ARE: AU‐rich element
BC: breast cancer
BCa: bladder cancer
BCL11A: B‐cell lymphoma/leukaemia 11A
BCLC: Barcelona Clinic Liver Cancer
CAS: Casticin
CC: cervical cancer
CML: chronic myeloid leukaemia
CRC: colorectal cancer
CRISPR: clustered regularly interspaced short palindromic repeats
CRPC: castration‐resistant prostate cancer
cryo‐EM: cryogenic electron microscopy
CSCC: cervical squamous cell carcinoma
CSCs: cancer stem cells
CSLCs: choriocarcinoma stem‐like cells
DAC: 5‐aza‐2‐deoxycytidine
DIM: 3, 3′‐diindolylmethane
DNC: dendrosomal nano‐curcumin
DNMT1: DNA methyltransferase 1
DNMTs: DNA methyltransferases
EC: Endometrial cancer
EMT: epithelial‐mesenchymal transitions
ENCODE: Encyclopaedia of DNA elements
ESCC: oesophageal squamous cell carcinoma
FANTOM: Functional Annotation of the Mammalian Genome
FDA: Food and Drug Administration
GBM: glioblastoma multiforme
GC: gastric cancer
GCs: granulosa cells
GI: gastrointestinal
GS: Gleason score
HBx: HBV X protein
HCC: Hepatocellular carcinoma
HDACs: histone deacetylases
HMTs: histone methyltransferases
HNSCC: Head and neck squamous cell carcinoma
HSCs: haematopoietic stem cells
HUCEC: healthy human cervical epithelial cell lines
IDO: indoleamine 2, 3‐dioxygenase
IFN: interferon
KLF4: Krüppel‐like factor 4
LCSCs: liver cancer stem cells
lincRNAs: long intergenic non‐coding RNA
lncRNAs: long non‐coding RNAs
LSC: leukaemia stem cell
LSCC: Laryngeal squamous cell carcinoma
LUAD: Lung adenocarcinoma
MBZ: Mebendazole
MEG3: maternally expressed gene 3
MIBC: muscle‐invasive bladder cancer
miRNAs: microRNAs
mRNA: messenger RNA
ncRNAs: non‐coding RNAs
NEAT1: nuclear paraspeckle assembly transcript 1
NMIBC: non‐muscle‐invasive bladder cancer
NPC: nasopharyngeal carcinoma
NPM‐ALK: nucleophosmin‐anaplastic lymphoma kinase
NSCLC: non‐small cell lung cancer
OC: ovarian cancer
OSLCs: osteosarcoma cancer stem‐like cells
PCa: prostate cancer
PCOS: polycystic ovary syndrome
PFS: progression‐free survival
Pol II: RNA polymerase II
PPI: Polyphyllin I
PRC2: polycomb repressive complex 2
PSA: prostate‐specific antigen
PTEN: phosphate and tensin homologue
PTGS: post‐transcriptional gene silencing
RCC: renal cell carcinoma
RFTS: Replication‐Foci Targeting Sequence
RUNX3: runt‐related transcription factor 3
SCLL: stem cell leukaemia/lymphoma
SNHG1: Small nucleolar RNA host gene 1
TILs: tumour‐infiltrating lymphocytes
TMZ: temozolomide
TNBC: triple‐negative breast cancer
TNM: tumour node metastasis
Topo II: topoisomerase II
TRD: target recognition domain
TSGs: tumour suppressor genes
UCCB: urothelial carcinoma of the bladder
UTF1: undifferentiated embryonic cell transcription factor‐1

## 1 Introduction

Cancer, in its essence, encompasses more than 100 distinct malignant diseases that manifest in different tissues throughout the human body [1, 2]. The elevated mortality rates linked to cancer are, in part, attributable to deficient early detection modalities and imprecise diagnostic instruments. Therefore, precise cancer diagnosis and prognosis estimation are crucial to improving patient survival rates. The prevailing cancer biomarkers, predominantly comprised of protein or peptide‐based entities like glycoproteins, often demonstrate fluctuations in their tissue or blood levels, serving as potential indicators for disease progression, including cancer [3].

An increasing body of research has substantiated the pivotal role of epigenetic alterations in tumorigenesis and cancer progression. Epigenetic processes are crucial for maintaining proper growth, development and gene control in various body systems [4]. When these mechanisms become disrupted, they can alter gene function, leading to pathological conditions such as cancer. So, tumorigenesis cannot be solely attributed to genetic modifications, as it also encompasses epigenetic transformations, including DNA methylation [5]. This covalent alteration can impede gene transcription by either obstructing the interaction between a transcription factor and its corresponding binding sites or recruiting methylated binding domain proteins that facilitate the suppression of gene expression [6].

*DNMT1* is an enzymatic catalyst that establishes DNA methylation patterns throughout cellular differentiation and development. *UHRF1* is a cofactor of *DNMT1* and binds directly to DNMT1 via its N‐terminal ubiquitin‐like domain (UBL). UHRF1 RING domain catalysed the binding of *DNMT1* to ubiquitinated histone H3, ensuring subnuclear localization of *DNMT1* and maintenance of DNA methylation [7]. Multiple investigations have demonstrated its pivotal contribution to the pathogenesis of cancer [8]. In this regard, Zhang et al. examined the correlation between *DNMT1* and aberrant methylation patterns of tumour suppressor genes (TSGs) and their association with the malignant phenotype observed in cervical cancer (CC). Their findings disclosed that the *DNMT1* methylation status could impact the activity of various crucial TSGs during the development of cervical tumours. Consequently, targeting *DNMT1* methylation holds promise as a viable therapeutic approach for treating CC [9]. Furthermore, *DNMT1*‐mediated effects in carcinogenesis may occur through the regulation of cell cycle‐ and apoptosis‐related genes. Notably, *DNMT1* silencing has been shown to increase Bax expression while decreasing *Bcl‐2* and *CCND1*/*2* in AN3CA cells, suggesting the potential of *DNMT1* in endometrial carcinoma (EC) therapy [10]. Thereby, among the numerous epigenetic regulators associated with cancer, DNMT1 has been identified as a key enzyme, owing to its fundamental role in maintaining cellular methyltransferase activity, regulating both global and gene‐specific demethylation, and the reactivation of TSGs in human cancer cells [11]. In this manner, exploring the function of DNMT1 in cancer presents a valuable opportunity to increase our understanding of tumour biology and to identify potential therapeutic targets.

Recent extensive research emphasises the importance of ncRNA molecules in governing the function of *DNMT1*. In this regard, DACOR1, a long non‐coding RNA (lncRNA), has been shown to activate tumour‐suppressor pathways and function as a regulator of cellular growth suppression. In terms of mechanism, DACOR1 markedly reduced the expression of cystathionine β‐synthase, a critical methyl donor in DNA methylation. Collectively, dysregulation of DNMT1‐associated lncRNAs plays a critical role in driving abnormal DNA methylation patterns and gene expression in colon tumorigenesis [12]. Furthermore, recent investigations offer valuable insights into the intercommunication and mechanisms involved in regulating *DNMT1* by ncRNA. This observation underscores the extensive engagement of ncRNAs and their interplay with crucial epigenetic modifiers, such as *DNMT1*, governing the expression of numerous target genes.

## 2 Noncoding RNA: From Biology to Functioning in Epigenetics

Human Genome Project Completion has unveiled that approximately 1.5% of the human genome is constituted by protein‐coding genes [13]. Indeed, the Encyclopaedia of DNA Elements (ENCODE) and the Functional Annotation of the Mammalian Genome (FANTOM), two prominent collaborative initiatives, have provided evidence indicating that a significant portion of the genome undergoes transcription and generates a diverse array of ncRNAs [14]. Presently, there is a prevailing belief that the level of intricacy exhibited by a species demonstrates a stronger correlation with the quantity of ncRNAs rather than the number of protein‐coding genes [15]. NcRNAs are indispensable agents in regulating essential cellular functions spanning all biological kingdoms. They actively govern diverse aspects of gene expression, including transcription and translation processes, thereby profoundly influencing genome organisation and stability [16]. Mounting evidence suggests that ncRNAs exert a diverse range of mechanisms, such as transcriptional processes, stability of messenger RNA (mRNA), post‐translational modifications, modulation of chromosome structure and RNA splicing. Notably, miRNA, lncRNA and circular RNA (circRNA) are among the extensively investigated ncRNAs. The subsequent section provides a more comprehensive elucidation of these well‐studied ncRNA types.

### 2.1 MiRNA

**FIGURE 1:**
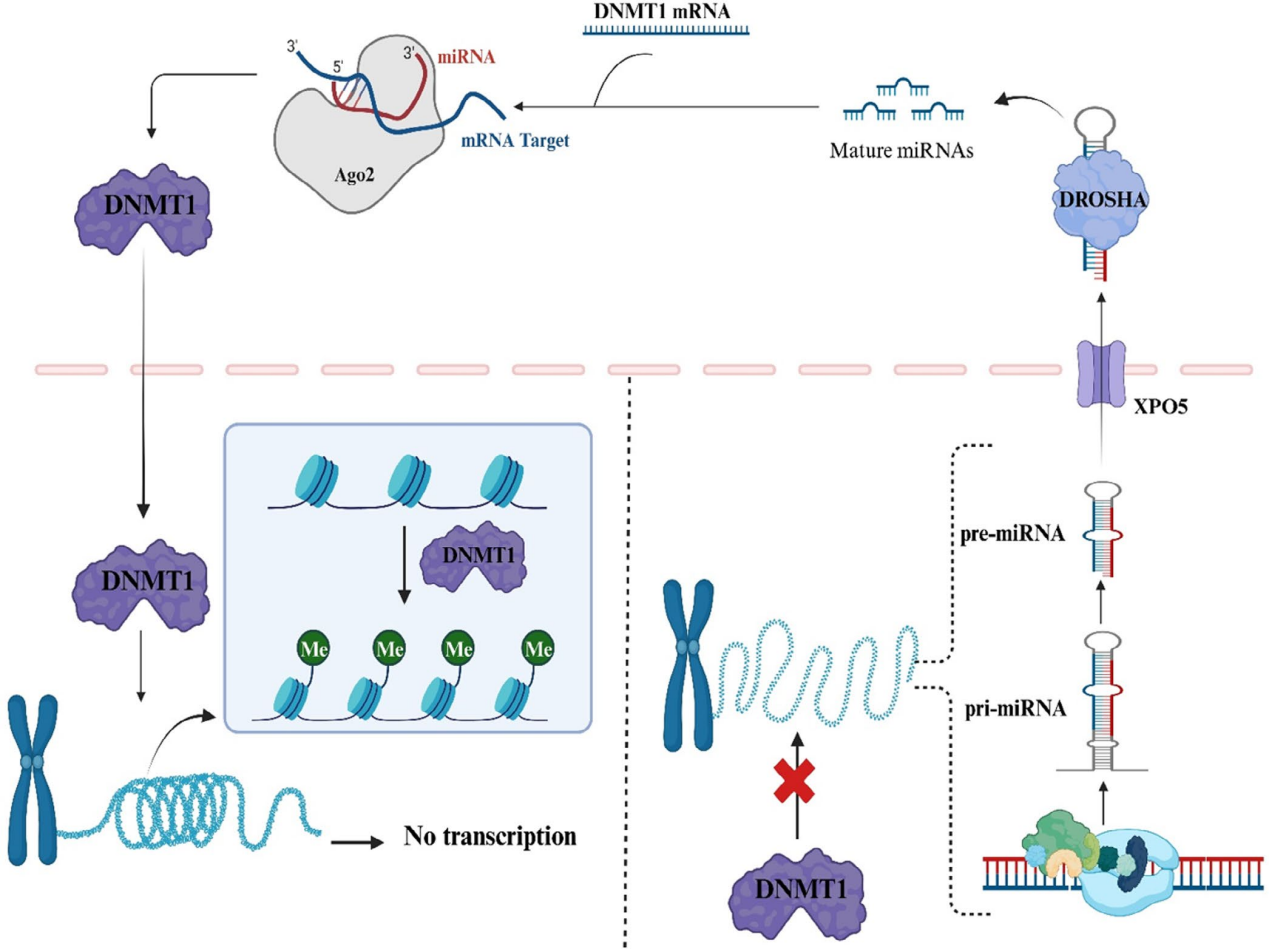
A schematic representation of the direct relationship between DNMT1 and miRNAs. The illustration portrays how specific miRNAs target DNMT1 mRNA, leading to the inhibition of its transcriptional activity. Conversely, DNMT1 exerts control by methylating the genes encoding these miRNAs, thereby impeding their own transcription. This bidirectional modulation highlights the intricate regulatory crosstalk between DNMT1 and miRNAs in epigenetic regulation.

MiRNAs represent a class of diminutive RNA molecules, typically about 22 nucleotides in length, which can exert negative post‐transcriptional regulation over their target gene expression [17]. *RNA polymerase II (Pol II)* transcribes these miRNAs into primary transcripts, which undergo processing within the cellular nucleus by the RNase III *Drosha* and *DGCR8* (microprocessor complex) to form precursor miRNAs [18]. Precursor miRNAs exhibit a configuration characterised by imperfect stem loops and undergo translocation to the cytoplasm facilitated by *Exportin‐5* [19]. Within the cytoplasmic compartment, these precursor miRNAs undergo additional processing by the RNase III Dicer to attain their ultimate functional mature miRNA form. MiRNAs exert their regulatory function by forming complexes with their target mRNAs, resulting in the downregulation of mRNA stabilities and translation. In cases where the miRNA exhibits complete complementarity with its target mRNA, it can initiate the degradation of the targeted mRNA molecule. MiRNAs can also engage with their targets through partial complementarity, frequently observed in the 3′ UTR regions of mRNAs. This interaction results in the translational suppression of the target genes by a partially understood mechanism that necessitates further investigation for complete elucidation [20]. Using post‐transcriptional gene silencing (PTGS) and mRNA degradation, miRNAs can govern the epigenome, thereby inducing downregulation of critical epigenetic modifiers and orchestrating alterations in the chromatin landscape [21]. Prominent instances of epigenetic factors engaging with miRNAs encompass histone deacetylases (*HDACs*), histone methyltransferases (*HMTs*) and DNA methyltransferases (*DNMTs*). Apart from the miRNAs that hold the capacity to regulate the epigenome, it is noteworthy that the expression of these miRNAs can, in turn, be subject to regulation through epigenetic modifications. For instance, CpG islands, typically prevalent at gene promoters, are likewise present in around half of all miRNA genes, rendering them susceptible to abnormal DNA methylation and consequent dysregulation of gene expression [22]. These epigenetic modifications can induce either the downregulation or upregulation of miRNA expressions and, these altered expression patterns have been linked to various stages of tumorigenesis. In this regard, Hu et al. conducted qRT‐PCR and genomic bisulfite sequencing to examine the epigenetic silencing of miR‐484 in CC. They observed that the insufficiency of *DNMT1*, which *EZH2* recruits, led to a decline in CpG methylation within the promoter region miR‐484, elevating miR‐484 expression levels. They concluded that miR‐484 was reduced due to *DNMT1*‐mediated hypermethylation occurring in its promoter region, and this molecular event contributes to its role as a tumour suppressor in CC [23]. These findings demonstrated a reciprocal relationship between *DNMT1* and miRNAs in human cancer (Figure 1).

### 2.2 LncRNA

LncRNAs, encompassing sequences exceeding 200 nucleotides, participate in many physiological and pathological processes, emphasising their significant involvement in cancer development [24]. LncRNAs exert regulatory control over tumour progression by actively engaging in gene expression, drug resistance and metastasis [25]. Remarkably, contemporary investigations have unveiled the multifaceted capacity of lncRNAs in orchestrating DNA methylation processes [12]. While the prevalence of this model remains uncertain in the present era, a diverse array of lncRNAs has been documented to engage *DNMTs* and govern the expression of target genes, thus assuming pivotal functions in various biological processes, including but not limited to osteoarthritis, neural differentiation, cardiovascular diseases, adipogenesis, mesoderm commitment, mental disorders, muscle regeneration and different cancer types [26]. Furthermore, certain lncRNAs have been demonstrated to act as sequestering agents for *DNMT*, thereby exerting a negative regulatory influence on DNA methylation. In this regard, nuclear paraspeckle assembly transcript 1 (*NEAT1*) directly interacts with *DNMT1*, leading to the subsequent suppression of P53 and cyclic GMP‐AMP synthase stimulator of interferon genes (*cGAS*/*STING*) expression in lung cancer. So, *NEAT1*, by interacting with DNMT1, inhibits the *cGAS/STING* pathway, thereby regulating cytotoxic T cell infiltration in lung cancer [27]. Furthermore, a recent functional investigation also substantiated the interaction between *lncRNA ATB* and *DNMT1*, stabilising *DNMT1* expression. Furthermore, *ATB* facilitated the association of *DNMT1* with *p53*. Importantly, heightened expression of *lncRNA ATB* expedited the proliferative and migratory capabilities of renal cell carcinoma (RCC) cells while concurrently hindering cell apoptosis. This effect is attributed to the *p53* reduction, which is facilitated by the binding of *ATB* to *DNMT1* [28]. Importantly, substantial evidence demonstrates that lncRNAs exert control over the expression of DNMTs and Ten‐Eleven Translocation enzymes (*TETs*) at various regulatory levels to modulate DNA methylation processes. Studies have reported that lncRNAs can suppress or promote DNMT expression, thus assuming crucial roles in cancer development. In this regard, *lncRNA GAS5* directly interacts with *EZH2*, consequently facilitating the assembly of the polycomb repressive complex 2 (*PRC2*). This molecular event, in turn, leads to the transcriptional suppression of *DNMT1* [29]. Therefore, lncRNAs, by regulating *DNMT1*, are involved in the epigenetic process.

## 3 DNMT1: From Biology to Functioning in Human Cancer

**FIGURE 2:**
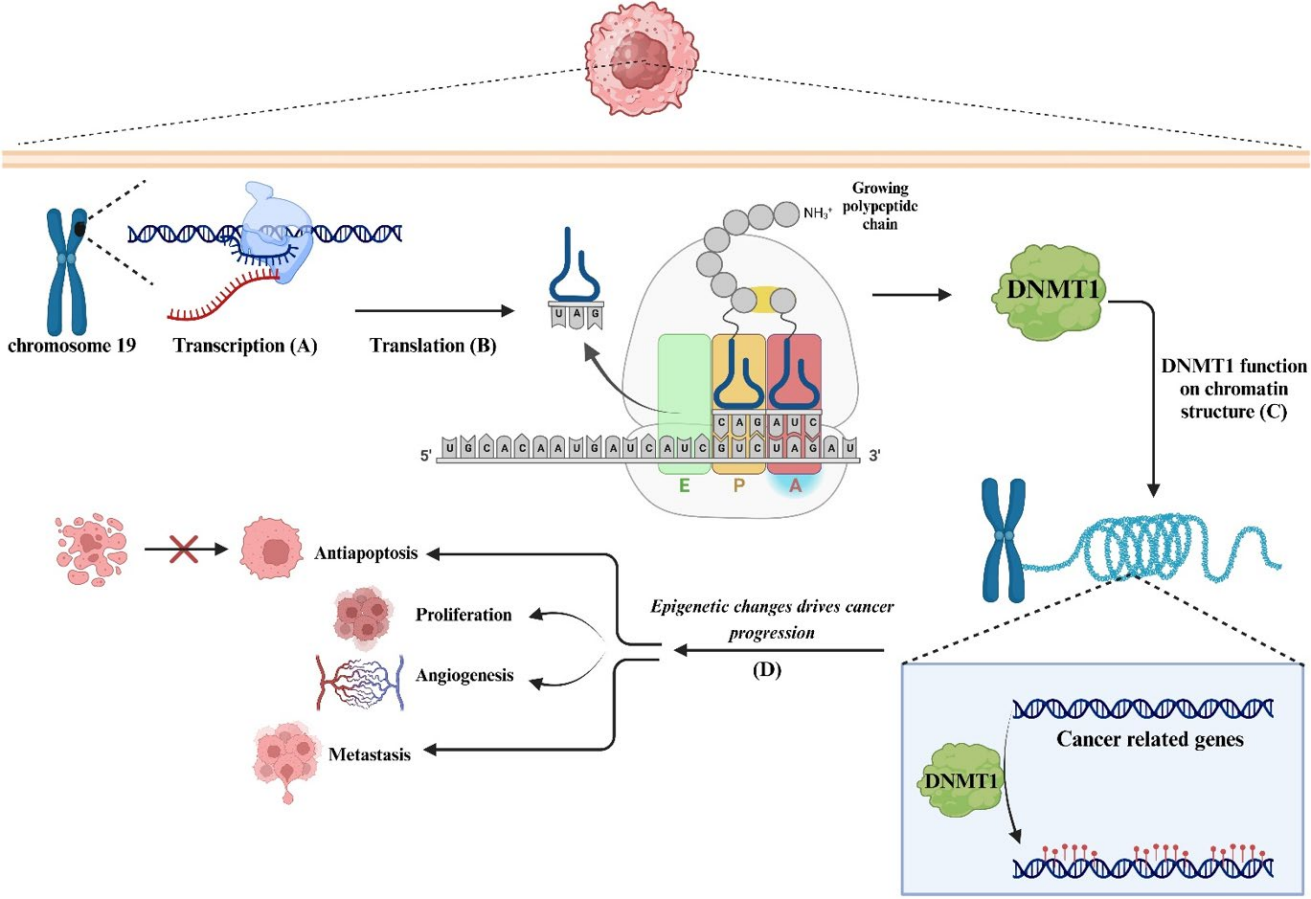
A schematic representation of DNMT1 location, expression and functioning in human cancer. (A) Depiction of DNMT1 transcriptional processes followed by (B) translation leading to its expression. (C) Highlighting DNMT1's functional impact on chromatin structure regulation. (D) Illustrating the downstream effects of epigenetic changes mediated by DNMT1 on key biological features of cancer cells, encompassing proliferation, apoptosis resistance, angiogenesis and metastasis. This comprehensive portrayal underscores the pivotal involvement of DNMT1 across multiple stages of cancer development and progression.

*DNMT1*, a considerable protein consisting of 1616 amino acids and featuring multiple domains, is intricately governed by intramolecular regulations that precisely restrict its functionality to hemimethylated DNA sites [30]. Notably, during DNA replication, *DNMT1* plays a pivotal role in propagating DNA methylation. *DNMT1* is classified as a class I methyltransferase family member, characterised by its possession of a conserved catalytic core known as the Rossmann fold. This core structure comprises a mixed seven‐stranded β‐sheet, bordered by three α‐helices on each side [31]. This enzyme facilitates the methylation reaction using an S‐adenosyl‐L‐methionine (AdoMet) —dependent mechanism. Within its catalytic core, it contains critical motifs responsible for both enzymatic catalysis and binding with the cofactor. An additional subdomain, the target recognition domain (TRD), is situated between the central β‐sheet and the final α‐helix of the catalytic core [32, 33]. Furthermore, extensive research spanning several decades has examined the structure and function of *DNMT1*. Kikuchi et al. explored the structural characteristics of human *DNMT1* (amino acid residues: 351–1616) through cryogenic electron microscopy (cryo‐EM). Their investigation involved the stimulation of *DNMT1* by the H3Ub2 tail and its formation of an intermediate complex alongside a hemimethylated DNA analogue. They present the cryo‐EM structure of the interaction between human *DNMT1* and its native co‐activators, namely hemimethylated DNA and ubiquitinated histone H3. They discover a previously unexplored linker positioned between the Replication‐Foci Targeting Sequence (RFTS) and CXXC domains, which serve as a critical mediator for activation. Concurrent with this phenomenon, there is a substantial reconfiguration of the inhibitory RFTS and CXXC domains, facilitating the enzyme to attain its complete functional capacity. The findings offer a basis for understanding how *DNMT1* is activated, which has implications for basic research and drug development [34]. Over the last 20 years, evidence has progressively linked the involvement and importance of *DNMT1* in tumorigenesis, aggressiveness and treatment response of human cancers. In this context, Liu et al. revealed that in breast cancer (BC), DNMT1‐mediated hypermethylation of the *FOXO3a* promoter results in the suppression of *FOXO3a expression*. *FOXO3a* exhibits functional interrelation with the repression of *FOXM1/SOX2* signalling, thereby leading to the consequential suppression of BCSC properties and tumorigenicity. Moreover, their investigation revealed that *SOX2* exerts direct transactivation on *DNMT1* expression, consequently inducing alterations in the methylation landscape. This, in turn, creates a feedback loop that leads to the inhibition of *FOXO3a* expression. Additionally, they unveiled that the suppression of DNMT activity resulted in the suppression of tumour growth by modulating the *FOXO3a/FOXM1/SOX2* signalling axis in BC. From a clinical perspective, a notable and statistically significant inverse relationship was observed between the expression levels of *FOXO3a* and *FOXM1/SOX2/DNMT1*. Furthermore, instances of diminished *FOXO3a* expression or elevated levels of *FOXM1*, *SOX2* and *DNMT1* were indicative of an unfavourable prognosis in BC patients. Their findings present compelling evidence regarding the significant involvement of the *DNMT1*/*FOXO3a*/*FOXM1*/*SOX2* pathway in regulating BCSC properties. This underscores the potential for identifying therapeutic targets for BC treatment based on these mechanistic insights [35]. Multiple studies have demonstrated that ncRNAs exhibit the ability to directly interact with *DNMT1*, resulting in alterations within the cancer cell's epigenome. This has the potential to reveal a previously unknown mechanism that accounts for the substantial alterations in the epigenome observed across different types of tumours. In this regard, *lncRNA KIF9‐AS1* is critical in regulating *RAI2* expression, mainly through the recruitment of *DNMT1* and subsequent modulation of *RAI2* DNA methylation. Additionally, upregulation of *RAI2* hindered the migration and proliferation while enhancing apoptosis in HCC cells. Further in vivo experimentation revealed that *KIF9‐AS1* silencing inhibits subcutaneous tumour formation. Thereby, *KIF9‐AS1* actively promotes HCC growth by facilitating *DNMT1*‐mediated promotion of *RAI2* DNA methylation [36](Figure 2).

## 4 LncRNAs/miRNA‐DNMT1 Axis in Cancer Pathogenesis

### 4.1 Hepatocellular Carcinoma

Hepatocellular carcinoma (HCC) represents the predominant form of primary liver malignancies globally, constituting approximately 90% of cases. This particular type of cancer stands as a prominent contributor to malignancy in humans, exhibiting substantial rates of both morbidity and mortality [37]. Therefore, a comprehensive understanding of the pathogenetic mechanisms underlying HCC and its regulatory processes is crucial for the effective management and treatment strategies employed for HCC.

#### 4.1.1 MiR‐152—DNMT1 Axis

*MiRNA‐148 (MiR‐148)* and *miR‐152* belong to the *miR‐148/152* family, which comprises *miR‐152*, *miR‐148 b* and *miR‐148a*. The members of this family may serve as valuable prognostic indicators and/or promising therapeutic targets for addressing diverse cancer types [38]. Recently, the functional importance of HBV X protein (HBx) in hepatocarcinogenesis has been explored. It was disclosed that *RIZ1* expression is significantly reduced within HCC tissues and is negatively regulated by *DNMT1* and recombinant HBV X protein (HBx). Also, *DNMT1* protein could bind to the promoter region of the *RIZ1* gene, and silencing *DNMT1* led to a decrease in the presence of methylated CpG sites within the genomic region associated with *RIZ1*. Notably, HBX recombinant plays a crucial role in *DNMT1* binding to the *RIZ1* gene promoter. Further mechanistic investigations demonstrated that the HBx upregulation led to a notable reduction in miR‐152 expression. Conversely, miR‐152 upregulation, primarily through direct targeting of *DNMT1*, resulted in the downregulation of *DNMT1* expression. So, HBx, by reducing miR‐152 expression, increases *DNMT1* expression. Significantly, this interplay between *miR‐152* and *DNMT1* has contributed, at least partially, to the epigenetic inactivation of *RIZ1*. Thereby, HBx primarily suppressed *RIZ1* expression in HCC by lowering *miR*‐*152* levels and increasing *DNMT1* levels, thus presenting a novel mechanism for the inactivation of *RIZ1* [39].

#### 4.1.2 MiR‐148a—DNMT1 Axis

*miR‐148a* functions as a miRNA with tumour‐suppressive properties, exerting a critical influence on the initiation and progression of HCC [40]. According to recent exploration, the silencing of miR‐148a in HCC cell lines is attributed to the hypermethylation of its CpG Island, and *DNMT1* upregulation serves as a causative factor behind the hypermethylation occurring at the *miR‐148a* promoter region. Interestingly, there is an inverse correlation between the expression of *DNMT1*, a target gene of *miR‐148a*, and the expression levels of *miR‐148a* within HCC cells. Significantly, *miR‐148a* upregulation markedly suppresses HCC cell cycle progression and cell proliferation. These results propose an innovative regulatory circuit involving *miR‐148a* and *DNMT1*, implying that *miR‐148a* functions as a tumour suppressor during hepatocellular carcinogenesis [41].

#### 4.1.3 MiR‐185—DNMT1 Axis

The differential expression of *miR‐185* has been observed to occur frequently in samples obtained from cancer patients. *MiR‐185* significantly downregulated in HCC tissues compared to the adjacent nonneoplastic liver parenchyma. Further, miR‐185 diminished in HCC cells as compared to primary hepatocytes. Functional experimentation disclosed that introducing exogenous *miR‐185* into HCC cells inhibited cellular proliferation and invasion in vitro and impeded tumour growth in *SCID* mice. Additionally, it was observed that *miR‐185* exhibits a direct targeting effect on *DNMT1* within HCC cells. Furthermore, upregulation of *miR‐185* reduced *DNMT1* protein levels in HCC cells. Significantly, the upregulation of *DNMT1* hindered the suppressive effects of *miR‐185* on HCC cell proliferation and invasion, thus implying the involvement of *DNMT1* in the inhibitory mechanism of *miR‐185* on HCC growth. Notably, the upregulation of *miR‐185* resulted in a decrease in *PTEN* promoter DNA methylation and an increase in *PTEN* expression, consequently leading to the suppression of Akt phosphorylation. However, the observed effects were somewhat counteracted by the upregulation of *DNMT1*. Thereby, *miR‐185* hinders the proliferation of HCC cells by selectively interacting with *DNMT1*, thereby inducing *PTEN* expression while inhibiting Akt activity [42].

#### 4.1.4 MiR‐378a‐3p—DNMT1 Axis

*MicroRNA‐378a*, comprising *miR‐378a‐3p* and *miR‐378a‐5p*, is derived from the *PPARGC1B* gene. It plays a critical role in tumour development and is an autonomous prognostic indicator for different types of malignant neoplasms [43]. *MiR‐378a* is considerably downregulated in HCC and corresponds to elevated microvascular density (MVD). Furthermore, the reduced expression of *miR‐378a‐3p* is a prognostic indicator for a diminished survival time among HCC patients. In addition, suppression of *miR‐378a‐3p* led to a noteworthy augmentation in vitro and in vivo angiogenesis. Furthermore, a direct association exists between miR‐378a‐3p and *TNF receptor‐associated factor 1 (TRAF1)*. This interaction led to the subsequent modulation of NF‐κB signalling, ultimately deregulating secreted *VEGF*. Mechanistic analysis unveiled that the downregulation of *miR‐378a‐3p* is attributed to the hypermethylation mediated by *DNMT1*. Moreover, *p65* instigated a positive feedback loop that enhanced the expression of *DNMT1*, thereby facilitating excessive methylation of the *miR‐378a‐3p* promoter region. In this manner, a positive feedback loop involving *DNMT1*, *miR‐378a‐3p*, *TRAF1* and *NF‐κB* plays a critical role in HCC cells, suggesting its potential as a viable therapeutic target for HCC [44].

#### 4.1.5 LncRNA‐GIHCG—DNMT1 Axis

The oncogenic potential of LncRNA GIHCG has been documented. It has been observed to exhibit upregulation and facilitate cellular proliferation and migration across various tumour types [45]. *LncRNA GIHCG* exhibited a gradual increase throughout the development of hepatocarcinogenesis and demonstrated a higher expression in HCC tissues when compared to adjacent non‐tumour tissues. Moreover, there is a significant association between elevated levels of *GIHCG* and larger tumour size, microvascular invasion, advanced Barcelona Clinic Liver Cancer (BCLC) stage, and unfavourable survival outcomes among HCC patients. Further experimental investigation demonstrated that *GIHCG* induces cellular proliferation and migration of HCC cells in vitro. Furthermore, *GIHCG* enhances xenograft tumour growth and metastatic potential in vivo. Further functional investigation revealed a direct physical interaction between *GIHCG* and *EZH2*, alongside their binding to the promoter regions of *miR‐200b/a/429*. Subsequently, this interaction recruits *EZH2* and *DNMT1* to the *miR‐200b/a/429* promoter sites, increasing histone H3K27 trimethylation and DNA methylation levels. Ultimately, these modifications lead to a significant downregulation of *miR‐200b/a/429* expression. Additionally, the physiological effects of *GIHCG* on HCC are contingent upon *miR‐200b/a/429* suppression. Collectively, *GIHCG/DNMT1/miR 200b/a/429* axis respective functions and operational mechanisms within HCC [46].

#### 4.1.6 LncRNA DDX11‐AS1—DNMT1 Axis

*DDX11‐AS1* on chromosome 12 exhibits oncogenic properties within HCC tissue specimens [47]. *DDX11‐AS1* expression is substantially upregulated in both HCC tissues and cell lines, with elevated *DDX11‐AS1* expression indicating unfavourable overall survival outcomes among patients. Functional analysis revealed that suppressing *DDX11‐AS1* hindered the proliferation, cell cycle advancement and migration of HCC cells, whereas its overexpression yielded contrasting outcomes. Furthermore, *DDX11‐AS1* exerts an inhibitory effect on *LATS2* expression in HCC cells. Significantly, *DDX11‐AS1* interacts with *EZH2* and *DNMT1*, thereby leading to the suppression of *LATS2* expression. Also, *DDX11‐AS1* silencing resulted in elevated levels of both mRNA and protein expression of *LATS2*. Conversely, the *LATS2* upregulation counteracted the stimulatory impact of *DDX11‐AS1* on cellular proliferation and invasion. Besides, in vivo experimentation revealed that *DDX11‐AS1* exerted a facilitative effect on tumour development, while the expression of *LATS2* mRNA displayed a substantial reduction within the tumour tissues and exhibited an inverse association with *DDX11‐AS1* expression. The *DDX11‐AS1/DNMT1/LATS2* pathway could serve as an oncogenic element in hepatocarcinogenesis, presenting a promising avenue for therapeutic intervention in treating HCC [48].

#### 4.1.7 LncRNA Linc‐GALH—DNMT1 Axis

*Linc‐GALH*, otherwise referred to as Gankyrin Associated lincRNA in HCC (*Linc‐GALH*), has been substantiated as an indispensable modulator of HCC. *Linc‐GALH* exhibited a significant level of expression that corresponded closely with the expression of Gankyrin in HCC. *Linc‐GALH* exhibited autonomous and unfavourable prognostic implications for HCC. Functional assays demonstrated that *Linc‐GALH* stimulated the migratory capabilities of HCC cells under in vitro conditions while concurrently augmenting the metastatic potential of HCC cells within the lungs in vivo. Notably, *linc‐GALH* expedites *DNMT1* degradation by augmenting ubiquitination, consequently facilitating the amplification of Gankyrin expression by reducing the methylation status specifically within HCC contexts. In this manner, *linc‐GALH* predominantly facilitates HCC cell migration by upregulating *Gankyrin* expression, achieved primarily via *DNMT1* degradation. Thereby, the *Linc‐GALH/DNMT1/Gankyrin* axis is both a prognostic biomarker and a viable therapeutic target for HCC that warrants investigation [49].

### 4.2 Gastric Cancer

Gastric cancer (GC) is a prevalent malignancy of the digestive system that exhibits a formidable prognosis, particularly among patients in advanced stages. The annual incidence of GC stands at approximately one million cases. Thus, discernment of innovative biomarkers and an enhanced comprehension of the mechanisms involved in GC carcinogenesis hold significant prominence [50].

#### 4.2.1 MiR‐148a—DNMT1 Axis

*MiR‐148a* has exhibited tumour‐suppressive properties in the context of GC. *MiR‐148a* exhibited abnormal downregulation in GC tissues and is comparatively lower in the MGC‐803 and HGC‐27 GC cell lines compared to the normal gastric epithelial cell line, GES‐1. Also, a significant association exists between reduced levels of *miR‐148a* and lymph node metastasis and tumour node metastasis (TNM) stage. Notably, overexpression of *miR‐148a* resulted in a notable decrease in the *cells'* in vitro migratory and invasive capabilities. Also, *DNMT1* serves as a direct and functional recipient of *miR‐148a*. Moreover, *miR‐148a* inhibitor led to amplified *DNMT1* expression within HGC‐27 cells, while upregulation of *miR‐148a* resulted in reduced *DNMT1* expression in MGC‐803 cells. Furthermore, overexpression of *DNMT1* effectively counteracted the suppressive effects exerted by *miR‐148a* on cellular migration. Taken together, *miR‐148a* exerts inhibitory effects on cellular migration in GC by modulating *DNMT1* activity [51]. Furthermore, *miR‐148a* could also function in GC pathogenesis via *MEG3*. *MEG3* is upregulated following the silencing of D*N*MT1 in GC cells. Additionally, inhibiting *MEG3* reduces the inhibitory effect on cell proliferation caused by the upregulation of *miR‐148a*. Notably, the inhibitory effect on *miR‐148a* might play a role in the decreased expression of *MEG3* in GC through the regulation of *DNMT1*. In this manner, the *miR‐148a/DNMT1/MEG3* axis exhibits promising potential as a therapeutic target for the treatment of GC [52]. In addition, Zuo et al. explored the involvement of miR‐148a and DNMTs in *RUNX3* promoter methylation and its subsequent impact on gene expression. It was observed that the expression of *RUNX3* mRNA exhibited a notable decrease in GC tissues as opposed to the corresponding normal tissues. Furthermore, this downregulation displayed a strong correlation with the expression of *miR‐148a*. A notable upregulation in the levels of *RUNX3* mRNA/protein and the unmethylated state of the *RUNX3* promoter was discerned after the administration of the DNA methylation inhibitor 5‐aza‐2′‐deoxycytidineto human GC AGS and BGC‐823 cells, as compared to cells that were not subjected to treatment. They additionally observed that the *miR‐148a* upregulation, a microRNA known to regulate *DNMT1* and *DNMT3B*, resulted in elevated levels of *RUNX3* expression within GC cells. They subsequently revealed that *DNMT1* silencing elevated *RUNX3* mRNA/protein levels, whereas *DNMT3B* silencing exhibited no discernible impact on these parameters within BGC‐823 cells. They also demonstrate the potential influence of *miR‐148a* on the regulation of *RUNX3* gene expression in GC, whereby it appears to modulate *DNMT1*‐mediated DNA methylation. These results shed light on a novel mechanism of gene expression regulation involving the interplay between microRNAs and epigenetic modification [53]. Importantly, Zhu et al. provided evidence indicating a consistent decrease in *miR‐148a* expression and heightened promoter region methylation in both GC tissues and cell lines. Inhibiting *DNMT1* expression reduced the methylation level of the *miR‐148a* promoter and subsequently facilitated the restoration of its expression. Also, excessive expression of *miR‐148a* in cancer cell lines led to a decline in *DNMT1* expression and hindered cell proliferation without any noticeable alteration in apoptosis rates. Moreover, DNA hypermethylation of the promoter region plays a role in the inactivation of *miR‐148a* in GC. Further, diminished expression of *miR‐148a* attenuates its inhibitory effect on *DNMT1* in GC, potentially leading to upregulated levels of *DNMT1* and facilitating DNA hypermethylation. Thereby, the *miR‐148a/DNMT1* axis is critically involved in the development of GC [54].

#### 4.2.2 MiR‐30b—DNMT1 Axis

*miR‐30b*, an endogenous miRNA derived from the gene on chromosome 8q24.22, exerts suppressive functions on cell proliferation and epithelial‐mesenchymal transitions (EMT) in various cancerous conditions [55]. *MiR‐30b‐5p* is significantly downregulated in GC specimens and associated with lymph node metastasis. *MiR‐30b‐5p* levels could be restored through DNA demethylation, while *DNMT1* induced *miR‐30b‐5p* promoter methylation. Further, functional experimentation suggested that the enforced expression of *miR‐30b‐5p* impacted cell migration, aligning with the tissue analysis findings. These discoveries offer an initial understanding of the epigenetic process underlying the downregulation of *miR‐30b‐5p*, facilitated by *DNMT1*. They shed light on the functional involvement of *miR‐30b‐5p* in the development of GC [56].

#### 4.2.3 MiR‐185—DNMT1 Axis

The chromosomal locus of miR‐185 is on chromosome 22, and it exerts tumour‐suppressive effects by modulating numerous pivotal biological processes including autophagy, apoptosis, EMT and the cell cycle of cancer cells [57]. Recent investigation disclosed that *GKN1* restitution exerted a suppressive effect on the proliferation of GC cells by instigating the production of endogenous miR‐185, which explicitly targets epigenetic regulators *DNMT1* and *EZH2* within the GC cells. *GKN1* ectopic expression resulted in *Tip60* overexpression and the *HDAC1* reduction in a *miR‐185*‐independent manner within GC cells. Consequently, this led to cell‐cycle arrest by modulating the expression of cell‐cycle proteins. Furthermore, an inverse relationship exists between the expression of *GKN1* and that of *DNMT1* and *EZH2* in a specific subgroup of GC. Interestingly, *GKN1* demonstrated a synergistic anti‐cancer effect when combined with 5‐fluorouracil in inhibiting tumour cell proliferation, thus implying a potential therapeutic approach for addressing GC. Therefore, the *GKN1/miR‐185/DNMT1* axis suppresses gastric carcinogenesis by controlling epigenetic modifications and cell cycle regulation [58].

#### 4.2.4 LncRNA HOTAIR—DNMT1 Axis

The lncRNA HOX transcript antisense RNA (*HOTAIR*) gene is located on chromosome 12q13.13. Extensive investigations have consistently demonstrated significant overexpression of *HOTAIR* in diverse types of human malignancies [59]. *HOTAIR* is upregulated, whereas *PCDH10* is reduced in GC. Depletion of *HOTAIR* resulted in a substantial increase in the mRNA/protein levels of *PCDH10* while concurrently reducing *PCDH10* methylation. Furthermore, *DNMT* expression substantially decreased upon *HOTAIR* silencing, while *HOTAIR* overexpression increased *DNMT1* expression. Further mechanistic analysis demonstrated an interaction between *miR‐148b* and *HOTAIR*. Moreover, *HOTAIR* silencing triggered *miR‐148b* overexpression, whereas the overexpression of *miR‐148b* had a corresponding downregulatory effect on *HOTAIR* expression. Furthermore, *HOTAIR* silencing and the introduction of *miR‐148b* mimic resulted in diminished *DNMT1* expression and *PCDH10* upregulation in GC. In this manner, *HOTAIR* interacts with *miR‐148b* and *DNMT1*, ultimately resulting in the methylation of *PCDH10*, thereby playing a role in the advancement of GC [60].

#### 4.2.5 LncRNA SNHG1—DNMT1 Axis

Small nucleolar RNA host gene 1 (*SNHG1*), situated on 11q12.3, is pivotal in the progression and prognostication of numerous cancer types [61]. *LncRNA‐SNHG1* expression is markedly elevated within GC tissues compared to adjacent tissues, positively associated with various clinicopathological parameters, including lymph node metastasis, T stage and TNM stage. So, patients with elevated levels of *lncRNA‐SNHG1* expression exhibited markedly reduced survival times compared to those with lower levels of expression. Further, *lncRNA‐SNHG1* significantly facilitated the proliferation of GC cells and enhanced DNMT1 expression. Therefore, *lncRNA SNHG1* enhances the expression of *DNMT1*, thereby fostering the process of GC cell proliferation [62].

#### 4.2.6 LncRNA SAMD12‐AS1—DNMT1 Axis in GC

*LncRNA SAMD12‐AS1* is derived from the antisense strand of the *SAMD12* gene, which is situated on human chromosome 8. It demonstrates dual roles as both a tumour suppressor and an oncogene [63]. *SAMD12‐AS1* exhibited substantial overexpression in both human GC tissues and cell lines compared to their normal counterparts. Elevated levels of *SAMD12‐AS1* expression are significantly associated with advanced TNM stage and reduced survival duration in individuals diagnosed with GC. *SAMD12‐AS1* augments the oncogenic potential of GC cells by impeding the P53 signalling pathway. Further functional examination revealed that *SAMD12‐AS1* potentially executes its biological functions in GC through direct interaction with *DNMT1*, thereby enhancing *DNMT1*‐mediated repression of the P53 signalling pathway. In this manner, *SAMD12‐AS1* contributes to the advancement of GC through the *DNMT1/P53* pathway [64].

### 4.3 Colorectal Cancer

Colorectal cancer (CRC), a prevalent neoplasm, ranks as the third leading contributor to mortality associated with cancer for both genders. Consequently, a comprehensive comprehension of the molecular mechanisms and pathways that drive CRC advancement is imperative, as it holds the potential for advancing innovative diagnostic techniques and targeted therapeutic interventions [65].

#### 4.3.1 MiR‐515—DNMT1 Axis

*MiR‐515–5p* is initially characterised as a miRNA specific to the placenta, playing a role in foetal development and growth. Several experimental studies have proposed that *miR‐515‐5p* exhibits tumour‐suppressive properties across different human cancers [66]. *Circ_0040809* and *DNMT1* expression are significantly upregulated, while *miR‐515‐5p* expression is downregulated in both CRC tissues and cells. Elevated levels of *circ_0040809* expression significantly correlate with decreased overall survival. Further experimentation disclosed that the suppression of *circ_0040809* impedes the proliferation and migration of CRC cells and encourages apoptosis. Conversely, the upregulation of *circ_0040809* yields contrasting effects. A mechanistic examination revealed that *circ_0040809* engages in competitive binding with *miR‐515‐5p*, resulting in an upregulation of *DNMT1* expression. Notably, partial attenuation of the tumour‐promoting effects mediated by *circ_0040809* can be observed through the overexpression of *miR‐515‐5p*. Thereby, *circ_0040809* enhances CRC cells' proliferative and migratory capabilities while impeding apoptosis by exerting regulatory control over the *miR‐515‐5p/DNMT1* pathway [67].

#### 4.3.2 MiR‐152—DNMT1 Axis

Wang et al. explored the impact of *DNMT1* on the biological properties of CRC cells. Their investigation revealed the presence of increased levels of *DNMT1* and *TMSB10* expression, diminished *miR‐152‐3p* expression and methylated *miR‐152‐3p* in both CRC tissues and cells. They observed that the downregulation of *DNMT1* or the upregulation of *miR‐152‐3p* resulted in a decrease in *TMSB10* expression, thereby exerting inhibitory effects on the progression of CRC and the growth of tumours. They additionally revealed that an increased expression of *DNMT1* could counteract the impact of *miR‐152‐3p* upregulation on the progression of CRC and the growth of tumours. They ultimately disclosed that *DNMT1* played a role in preserving the methylation pattern of *miR‐152‐3p*, and *miR‐152*, in turn, directly targets *TMSB10*. Thereby, inhibition of *DNMT1* leads to the absence of methylation in *miR‐152‐3p*, causing a reduction in *TMSB10* expression and subsequently impeding CRC progression [68].

#### 4.3.3 LncRNA LINC00337—DNMT1 Axis

Recent in silico analysis detects promoter region methylation of *CNN1* in CRC. In vitro investigations revealed hypermethylation of the *CNN1* promoter region, specifically in the context of CRC, which is associated with reduced *CNN1* expression in both CRC tissues and cells. Further mechanistic investigation provided compelling evidence that *LINC00337* facilitated the recruitment of *DNMT1* to the promoter region of *CNN1*, thereby exerting transcriptional repression on *CNN1*. These findings demonstrate that hypermethylation of the *CNN1* promoter region in CRC is associated with increased expression of *LINC0033*. Further functional analyses showed that the upregulation of *CNN1* or *LINC00337* silencing impeded CRC cell proliferation, migration/invasion and proangiogenetic activity in vitro. These findings were further supported by in vivo experiments, which demonstrated enhanced tumour growth, increased MVD and increased levels of *VEGF* and *Ki67*. *LINC00337* promotes the development of tumours and angiogenesis in CRC by recruiting *DNMT1* to suppress *CNN1* [69].

### 4.4 Pancreatic Cancer

Pancreatic cancer represents a highly malignant neoplasm of the digestive system, displaying a poor prognosis. The majority of individuals who have pancreatic cancer receive their diagnosis during advanced stages or even when metastasis has occurred, owing to its remarkably aggressive nature and absence of discernible early symptoms. Thus, timely detection of pancreatic cancer plays a pivotal role in enhancing its prognosis [70].

#### 4.4.1 MiR‐34a—DNMT1 Axis in Pancreatic Cancer

*miR‐34a*, belonging to the *miR‐34* family, is situated on chromosome 1p36. It is recognised as a pivotal controller of tumour suppression. Consequently, trials have been undertaken to explore the clinical use of miR‐34a replacement, marking it as the initial endeavour in utilising miRNA for cancer therapy [71]. According to recent experimentation in pancreatic cancer, *DNMT1* exerts repressive effects on *miR‐34a* expression while concurrently promoting the activation of the Notch pathway through mediation of the hypermethylation process targeting the *miR‐34a* promoter region. An inverse correlation is observed between the expression levels of *DNMT1* and *miR‐34a* in individuals diagnosed with pancreatic cancer. Mechanistically, *DNMT1* silencing reduced methylation levels at the promoter region of *miR‐34a*, leading to an upregulation of *miR‐34a* expression. Consequently, this upregulation exerted inhibitory effects on the Notch pathway activity. Furthermore, attenuation of the Notch signalling pathway through the *DNMT1*/*miR‐34a* axis substantially augmented the susceptibility of pancreatic cells towards molecular targeting agents. Thereby, downregulation of DNMT exhibits a stimulatory effect on the expression of *miR‐34a*, highlighting its prospective utility as a therapeutic target for pancreatic cancer [72].

#### 4.4.2 MiR‐152—DNMT1 Axis

In pancreatic cancer, increased expression of *DNMT1* and aberrant methylation of promoters implicated in the downregulation of *KLF4* expression result in impaired differentiation and unfavourable outcomes. Also, modulation of *KLF4* expression substantially impacts the expressions of differentiation markers in cells afflicted with pancreatic cancer. In addition, administration of 3, 3′‐diindolylmethane (DIM) through the diet substantially stimulates the expression of *miR‐152*. Consequently, this upregulation hinders the expression of *DNMT1* protein and its interaction with the promoter region of *KLF4*, resulting in a decrease in promoter DNA methylation and the activation of *KLF4* expression within pancreatic cancer cells. Notably, administration of DIM results in notable suppression of cellular proliferation in vitro and the inhibition of tumour formation in animal models of pancreatic cancer. In this manner, induction of the *miR‐152/DNMT1/KLF4* signalling pathway through epigenetic mechanisms by dietary DIM leads to the differentiation and substantial growth suppression of pancreatic cancer cells. This finding underscores its potential translational relevance for pancreatic cancer and other malignancies [73].

#### 4.4.3 MiR‐148a/b‐DNMT1 Axis

According to recent experiments, there is mutual influence between *miR‐148a* and *DNMT1*, which could impact cellular proliferation and migration in pancreatic cancer cells. Accordingly, restoration of miR‐148a resulted in the reactivation of TSGs, including *p16*, *preproenkephalin* and *Ras association domain family member 1* in the AsPC‐1 pancreatic cancer cell line by specifically targeting *DNMT1*. Importantly, upregulation of *miR‐148a* significantly inhibits cell proliferation and migration in AsPC‐1 cells. Thereby, targeting *miR‐148a/DNMT1* could be a promising therapeutic strategy for managing pancreatic cancer [74]. *p27* is another gene in which miR‐148a could suppress pancreatic cancer advancement. In this regard, it was disclosed that overexpression of miR‐148a by suppressing DNMT1 hindered the methylation process of *p27*, resulting in an elevated expression of *p27*. This is associated with attenuated proliferative and metastatic capacities of ASPC‐1 cells. Interestingly, the suppression of *DNMT1* resulted in *miR‐148a* upregulation. Notably, in vivo investigations provided substantial evidence for effectively suppressing ASPC‐1 tumorigenesis through upregulating *miR‐148a* or *DNMT1* silencing [75]. Furthermore, *miR‐148b* and‐152, by regulating the expression of *SPARC* and *BNIP3*, play a crucial role in pancreatic cancer pathogenesis. It was disclosed that upregulation of *miR‐148b* and‐*152* led to the restoration of DNA methylation patterns to their normal states and facilitated TSGs re‐expression, such as *SPARC* and *BNIP3* in pancreatic cancer cell lines (AsPC‐1 and MIA PaCa‐2). In summary, miRs that specifically target *DNMT1* and modulate the methylation patterns of TSGs like *BNIP3* and *SPARC* can potentially be utilised as a therapeutic approach for inducing apoptosis in pancreatic cancer cells and reducing their tumorigenic properties [76].

#### 4.4.4 MiR‐377‐DNMT1 Axis

*MiR‐377*, an RNA molecule synthesised by the 14q32 miRNA cluster, has been identified as a key contributor to the pathogenesis of diverse malignancies, including pancreatic cancer [77]. A reciprocal relationship exists between *miR‐377* and *DNMT1* in pancreatic cancer cells, where *DNMT1*‐mediated promoter methylation significantly influences miR‐377 expression, while *DNMT1* itself functions as a downstream target of *miR‐377*. In tumour specimens, a discernible presence of hypermethylation in the promoters of *PENK*, *TFPI2*, *SPARC* and *BNIP3* was observed, whereas normal tissues exhibited no such methylation patterns. Notably, *miR*‐*377* exhibited substantial suppressive effects on cellular proliferation while triggering apoptosis. Therefore, in pancreatic cancer cells, the modulation of *miR‐377*, specifically by targeting *DNMT1*, can potentially diminish DNA methylation levels associated with specific TSGs, thereby facilitating the reinstatement of their expression [78].

### 4.5 Haematological Cancer

Haematological malignancies encompass malignant neoplasms arising from the aberrant differentiation of haematopoietic stem cells (HSCs). Researchers are compelled to explore innovative treatment targets and mechanisms due to the prevalent systemic engagement, unfavourable prognosis, chemoresistance and frequent recurrence observed in haematological malignancies [79].

#### 4.5.1 MiR‐148a‐DNMT1 Axis

In acute myeloid leukaemia (AML) patients, *miR‐148a* expression was significantly downregulated, while DNMT1 was upregulated. The methylation status of the *miR‐148* promoter markedly increased in AML cell lines, highlighting the underlying cause for the decreased expression of *miR‐148a* in both AML patients and cell lines. In contrast, silencing *DNMT1* significantly reduces the methylation level of the *miR‐148a* promoter, resulting in a substantial upregulation of *miR‐148a* expression. Subsequent experimentation demonstrated that *miR‐148a* exerts direct negative regulatory control over *DNMT1*, and overexpression of *miR‐148a* decreased expression levels of *DNMT1* in terms of mRNA/protein. Conversely, silencing *miR‐148a* in Kasumi‐1 cells led to an elevation in *DNMT1* expression levels. Further cellular analysis showed that elevated expression of *miR‐148a* suppressed cellular proliferation while simultaneously fostering apoptosis. In this manner, these findings indicate the existence of a reciprocal negative feedback loop between *miR‐148a* and *DNMT1* in the context of AML [80].

#### 4.5.2 MiR‐152—DNMT1 Axis in Non‐Hodgkin Lymphoma

Silencing *DNMT1* significantly increases the expression of TSGs (*SHP‐1, p14, p16, BCL2L10* and *SOCS3*) by reducing their methylation levels in OCI‐Ly10 and Granta‐159 cells. At the cellular level, suppression of *DNMT1* hinders the cellular proliferation, formation of cell colonies and progression of the cell cycle while also triggering apoptosis in lymphoma cells. Furthermore, *miR‐152* exerts its downregulatory effect on *DNMT1* expression by directly targeting the gene, and *miR‐152* overexpression results in elevated expression levels of TSGs, specifically *SHP‐1* and *SOCS3*. Additionally, *miR‐152* induces apoptosis and impedes cell proliferation. Moreover, the upregulation of *miR‐152* has a profound inhibitory effect on tumour development in vivo, as evidenced by a reduction in *DNMT1* expression and an augmentation in the expression of TSGs. In this manner, *miR‐152* exerts an inhibitory effect on lymphoma growth through its capacity to suppress the *DNMT1*‐mediated silencing mechanism of *SOCS3* and *SHP‐1* [81].

#### 4.5.3 LncRNA HOTAIR—DNMT1 Axis

Recent exploration disclosed that the expressions of *HOTAIR* and *DNMT1* elevated, whereas *PTEN* demonstrated decreased expression in both CML cells and the bone marrow of CML patients. *HOTAIR* predominantly engaged in molecular interactions with *DNMT1*, while *DNMT1* primarily exhibited binding affinity towards the promoter region of *PTEN*. So, *HOTAIR*, by binding with *DNMT1*, modulates *PTEN* promoter methylation. Additionally, depletion of *HOTAIR* or *DNMT1* resulted in diminished migration, colony formation, proliferation and increased apoptosis rate of CML cells. Furthermore, reduced expression of *HOTAIR* and *DNMT1* led to decreased tumour volume and weight in mice injected with CML cells. Thereby, a reduction in *HOTAIR* levels inhibits its association with *DNMT1*, consequently impeding the growth of CML cells and promoting programmed cell death. This phenomenon is intricately linked to the control of *PTEN* promoter methylation [82].

#### 4.5.4 LINC00173—DNMT1 Axis

Dysregulation of *LINC00173*, an intergenic noncoding RNA positioned at chromosome 12q24.22, has been highlighted in various human cancer types [83]. *LINC00173* displays decreased expression levels, while *DNMT1* increases in AML. Additionally, they discovered a negative correlation exists between the methylation of the *LINC00173* promoter and its expression. These findings were consistent across multiple databases, including GEPIA and CCLE and in various contexts, such as benzene‐exposed workers, B‐cell non‐Hodgkin's lymphoma and HQ‐induced malignantly transformed TK6 cells (HQ‐MT cells). Mechanistic investigation disclosed that depletion of *DNMT1* led to diminished *LINC00173* promoter methylation in HQ‐MT cells. Furthermore, *LINC00173* upregulation suppressed *DNMT1* expression while inhibiting cell proliferation and tumour growth in HQ‐MT cells. Additionally, this overexpression increased responsiveness to cisplatin chemotherapy and promoted apoptosis. Notably, an interaction between *LINC00173* and *DNMT1* takes place to exert control over the methylation process of the LINC00173 promoter region. Overall, the interplay between *DNMT1* and *LINC00173* governs the modulation of *LINC00173* expression via regulation of its promoter methylation level. This, in turn, modulates the functioning of HQ‐MT cells both in vitro and in vivo, thereby presenting a novel therapeutic target for benzene‐induced tumours [84].

### 4.6 Ovarian Cancer

Ovarian cancer (OC) is an exceptionally aggressive malignancy that significantly endangers the well‐being of women and presents formidable obstacles for healthcare practitioners. On a global scale, this malignancy ranks as the seventh most prevalent form of cancer and the eighth primary contributor to mortality among women who have cancer. Thus, the global health burden of OC necessitates immediate attention to molecular investigations that offer novel approaches to enhancing disease prognosis [85].

#### 4.6.1 Lnc‐MAP3K13–7:1—DNMT1 Axis

Geng and colleagues explored the contribution of *DNMT1* in the pathophysiology of polycystic ovary syndrome (PCOS). They noted a significant upregulation of *lnc‐MAP3K13–7:1* in granulosa cells (GCs) from individuals diagnosed with PCOS. This was accompanied by a concurrent decrease in global DNA methylation levels, reduced expression of *DNMT1* and elevated levels of *cyclin‐dependent kinase inhibitor 1A* (*CDKN1A*, *p21*) expression. They observed that the upregulation of *lnc‐MAP3K13–7:1* in KGN cells led to a halt in cell cycle progression, specifically in the G0/G1 phase. Additionally, it resulted in the suppression of *DNMT1* at the molecular level. Their mechanistic investigation unveiled that *lnc‐MAP3K13–7:1*, through its role as a protein‐binding scaffold, effectively suppressed the expression of *DNMT1* and led to ubiquitin‐mediated degradation of the DNMT1 protein. *DNMT1*‐dependent *CDKN1A* promoter hypomethylation also increased *CDKN1A* transcription, inhibiting GC growth. In this manner, *lnc‐MAP3K13–7:1*‐dependent inhibition of *DNMT1* controls the expression of *CDKN1A*/*p21* and impedes the proliferation of GC cells [86].

### 4.7 Breast Cancer

Breast cancer is the prevailing malignancy among women, and despite therapeutic advancements, it remains the primary contributor to cancer‐related mortality in females on a global scale. The limited range of therapeutic interventions for BC can be attributed to the prevalent manifestation of chemoresistance. Consequently, there is a need to unravel the fundamental molecular mechanisms underlying this pathology and advance novel approaches to managing this disease [87].

#### 4.7.1 MiR‐497—DNMT1 Axis

*miR‐497*, an extensively preserved microRNA transcribed from the initial intron of the *MIR497HG* (Gene ID: 100506755) gene situated on the 17p13.1 locus of the human chromosome, is a member of the miR‐15 family. *MiR‐497* reduction has been evident in diverse carcinoma types, such as BC, thereby implying the potential tumour‐suppressive function of miR‐497 [88]. The *miR‐497/GPRC5A* axis, a recently identified mediator of BC, could be regulated through *DNMT1*. In the context of *BC*, *DNMT1* is significantly increased, while the expression of *GPRC5A* is reduced. Overexpression of *DNMT1* significantly enhances both resistance to chemotherapy and the metastatic potential of BC. *DNMT1* induces modifications in the methylation status of the CpG island located within the promoter region of *miR‐497*, consequently repressing *miR‐497* expression. Notably, *miR‐497* exhibited a specific affinity for inhibiting *GPRC5A* expression, thereby impeding chemotherapy resistance and suppressing the metastatic potential of BC cells. In this manner, *DNMT1* potentially obstructs *miR‐497* and enhances the activation of *GPRC5A* via methylation, thereby intensifying the resistance to chemotherapy and metastasis in BC [89].

#### 4.7.2 MiR‐152/148a—DNMT1 Axis

Sengupta et al. explored the interrelationship between *miR‐152*, *DNMT1* and *CDH1* activity concerning BC's metastatic potential and aggressiveness. They noticed that *miR‐152* directly regulates *DNMT1* in the MDA‐MB‐231 cell line. They confirmed a correlation between elevated expression of *DNMT1* and gene hypermethylation, which subsequently triggers *miR‐152* gene repression. They subsequently unveiled the pivotal involvement of *DNMT1* in governing the regulatory mechanisms of the *miR‐152* gene. They noticed that inhibition of *DNMT1* protein activity leads to the dominance of *miR‐152* expression, resulting in the degradation of *DNMT1* mRNA. This intricate regulatory mechanism forms a recurring feedback loop, currently being investigated as the *DNMT1/miR‐152* switch for controlling the activation and deactivation of target genes regulated by *DNMT1*. Their investigation yielded the identification of a regulatory mechanism wherein the *DNMT1/miR‐152* switch exerts influence over the modulation of *CDH1* gene expression. In addition, silencing *DNMT1*, which leads to the upregulation of *CDH1*, also known as the *DNMT1/CDH1* loop, in the presence of excessive ectopic expression of *miR‐152* effectively inhibits the migratory capability of cancer cells. Thus, the interplay of *miR‐152*, *DNMT1* and *CDH1* signifies a pivotal involvement in BC metastasis [90]. In addition, Xu et al. observed that *miR‐148a* and *miR‐152* expression levels exhibit a reduction in BC tissues and cells owing to CpG island hypermethylation. Next, they observed an abnormal increase in the expression of *DNMT1* in BC, and this heightened expression is the primary cause of excessive methylation observed in the promoters of *miR‐148a* and *miR‐152*. Intriguingly, an inverse correlation exists between the expression levels of *miR‐148a/152* and *DNMT1*, a target gene impacted by *miR‐148a/152*. Their outcomes led them to put forward a hypothesis suggesting the existence of a negative feedback regulatory loop between *miR‐148a/152* and *DNMT1* in the context of BC. More importantly, they disclosed that *miR‐148a* and *miR‐152* effectively target the proteins *IGF*‐IR and *IRS1*, which are frequently upregulated in BC. The overexpression of either *miR‐148a* or *miR‐152* significantly inhibits BC tumour angiogenesis, colony formation and cell proliferation. This inhibitory effect is achieved by targeting *IGF‐IR* and *IRS1*, suppressing the downstream signalling pathways of AKT and MAPK/ERK. In this manner, their findings propose an innovative regulatory pathway involving *miR‐148a/152* and *DNMT1*, highlighting the tumour suppressive roles of *miR‐148a* and *miR‐152* by targeting *IGF‐IR* and *IRS1* [91].

#### 4.7.3 MiR‐185—DNMT1 Axis

*miR‐185* significantly decreased within both triple‐negative breast cancer (TNBC) tissues and cell lines and correlated with various clinical factors, including overall survival, clinical stage, lymph node metastasis and relapse‐free survival in TNBC. Additionally, aberrant *miR‐185* ectopic expression suppressed TNBC cell proliferation both in vivo and in vitro. Furthermore, *miR‐185* exhibited direct binding specificity towards *DNMT1* and *E2F6*, leading to a substantial upregulation of *BRCA1* expression at both the mRNA and protein levels in TNBC. Therefore, *miR‐185* plays a role in inhibiting tumour growth during the development of TNBC, suggesting the *miR‐185/DNMT1* axis is a promising therapeutic approach for TNBC [92].

#### 4.7.4 MiR‐142‐5p—DNMT1 Axis

The *miR‐142* gene at the chromosomal locus 17q22 plays a significant role in modulating cellular migration, proliferation and apoptotic processes across various malignancies [93]. Myocardin‐related transcription factor A (*MKL*‐1) can adhere to the conserved cis‐regulatory element CC (A/T) 6GG, commonly referred to as the CarG box, situated within the *miR‐142‐5p* promoter region. This interaction facilitates the regulation of *miR‐142‐5p* transcription. Furthermore, experimental evidence demonstrated that *miR‐142‐5p* directly targets the 3′‐UTR region of *DNMT1*, suppressing *DNMT1* expression. As a result, a feedback loop is established, hindering BC cell migration and proliferation. So, their study offers significant and innovative contributions to understanding the *MKL‐1/miR‐142‐5p/DNMT1/maspin* signalling pathway, potentially serving as a novel concept for BC's diagnosis, treatment and prognosis [94].

#### 4.7.5 LncRNA H19—DNMT1 Axis in BC

*LncRNA H19*, an early‐identified lncRNA, is situated within the genomic vicinity of chromosome 11p15.5. The aberrant upregulation of *H19* is widely believed to be implicated in the oncogenesis and advancement of various cancers across different anatomical systems in the human body, including the breast [95]. *H19* significantly upregulated in both human breast tumour tissues and cells. H19 displays an inverse association with the expression levels of *miR‐152*, while a direct relationship exists between the expression levels of *H19* and *DNMT1* mRNA. Mechanistically, *H19* functions as an endogenous sponge by directly associating with *miR‐152*, while *miR‐152* specifically regulates *DNMT1*. So, the upregulation of *H19* significantly alleviated the inhibitory effects of *miR‐152* on the expression of *DNMT1*. Furthermore, the pronounced antagonistic impacts of *H19* downregulation on cellular proliferation and invasion are effectively counteracted by the inhibition *miR‐152* and the enhanced expression of *DNMT1*. In conclusion, *H19* facilitated the proliferation and invasion of BC via the *miR‐152/DNMT1* axis, presenting a novel explanatory mechanism elucidating the pathogenesis and progression of BC [96].

### 4.8 Endometrial Cancer

EC is categorised as a gynecologic malignancy and ranks as the sixth most prevalent tumour among females. The present therapeutic strategies for EC encompass chemotherapy, radiotherapy, brachytherapy and surgical excision. Despite significant advancements in the therapeutic domain concerning EC within the last few years, the prognosis for EC remains unfavourable. Consequently, it is imperative to delve into the molecular mechanisms that facilitate the advancement of EC to enhance the efficacy of its treatment [97].

#### 4.8.1 MiR‐148a/b—DNMT1 Axis

*miR‐148b*‐mediated regulation of *DNMT1* has been identified as a key factor in the pathogenesis of EC. In this context, the expression of *miR‐148b* greatly decreased in both EC tissues and HEC‐1A and HEC‐1B cells, while *DNMT1* exhibited elevated expression levels. Overexpression of *miR‐148b* resulted in the suppression of cellular proliferation and hindered the progression of the cell cycle while concurrently promoting cellular apoptosis. In EC cells, it was ascertained that *DNMT1* functions as a target gene of *miR‐148b*. Thereby, *miR‐148b* exerts an inhibitory effect on cellular proliferation and promotes apoptotic processes in EC by modulating the activity of *DNMT1* [98]. Additionally, *miR‐148b* plays a crucial role in hypoxic stress‐mediated epigenetic modifications in the pathogenesis of EC. In this vein, *DNMT1* protein expression decreased within ectopic endometriotic stromal cells compared to eutopic endometrial stromal cells, and exposure to hypoxia resulted in a substantial downregulation of *DNMT1* levels. Also, there is a direct targeting of *DNMT1* by *miR‐148a*, establishing evidence for the hypothesis that the decreased expression of *DNMT1* in ectopic endometriotic stromal cells could be attributed to elevated levels of *miR‐148a*. Furthermore, hypoxia diminishes the presence of HuR protein and its interaction with the AU‐rich element (ARE) situated at the 3′‐UTR of *DNMT1* transcript, consequently resulting in the heightened affinity of AUF1 to the aforementioned ARE. This observation substantiates the hypothesis positing competitive engagement between AUF1 and HuR. Additionally, AUF1 binding to the ARE facilitates recruitment of the *miR‐148a*‐AGO2 complex to the adjacent *miR‐148a* binding site, thereby shedding light on the mechanism by which AUF1 binding leads to diminished mRNA stability. The researchers' discovery is substantiated by a recent report indicating that AUF1 assists in recruiting miRNA‐loaded AGO2 to specific mRNA targets. Accordingly, these findings illustrate the influence of the interplay between AUF1 and HuR on the effectiveness of miR‐148a targeting. Consequently, this intricate relationship is significant in governing the regulation of *DNMT1* expression and DNA methylation in response to hypoxic stress conditions. Therefore, microenvironmental hypoxia plays a vital role in suppressing *DNMT1* through the involvement of *AUF1*/*miR‐148a*. Consequently, the downregulation of *DNMT1* leads to epigenetic modifications. Therefore, manipulating the interactions among *AUF1*, *miR‐148a* and the transcript of *DNMT1* holds potential for future development in restoring *DNMT1* expression [99].

### 4.9 Prostate Cancer

Prostate cancer (PCa) is a complex condition that arises from various causal factors, encompassing epigenetic modifications and genomic changes. Although the conventional diagnostic criterion for PCa involves assessing the prostate‐specific antigen (PSA) in the bloodstream, it is noteworthy that this biomarker may also exhibit elevated levels in other prostate‐related ailments, such as prostatitis and benign prostatic hyperplasia. Moreover, no apparent correlation exists between the PSA level and the PCa stage. To effectively manage PCa in a clinical setting, it is imperative to identify novel and dependable biomarkers that offer therapeutic, prognostic and diagnostic insights [100].

#### 4.9.1 MiR‐148/152—DNMT1 Axis

*DNMT1* is a significant driver of *miR‐148a*‐mediated biological processes in the pathogenesis of PCa. In this context, in silico analysis of miRNA target prediction indicated that *miR‐148a* exhibits a binding affinity towards the 3′ UTR of *DNMT1* mRNA, potentially inducing *DNMT1* gene silencing. Furthermore, *miR‐148a* ectopic expression triggers apoptosis and impedes cellular proliferation by suppressing *DNMT1*. Moreover, a regulatory relationship exists between DNA methylation, *DNMT1*, and the *miR‐148a* gene in the context of PCa. So, DNA methylation plays a role in the suppression of miR‐148a. In contrast, overexpression of *miR‐148a* leads to the downregulation of *DNMT1* expression and the activation of apoptotic genes in hormone‐refractory prostate cancer cells [101]. In addition, *DNMT1* could also exert its effects on PCa pathogenesis through the regulation of *miR‐152*. In this regard, *miR‐152* exhibits significant differential expression in prostate cancer cell lines AA and CA and is markedly downregulated in the more aggressive cells. However, 5‐aza‐2′‐deoxycytidine an inhibitor of *DNMT1*, significantly reduced the methylation status of the *miR‐152* promoter, resulting in increased expression of *miR‐152*. Furthermore, they observed that the overexpression of *miR‐152* led to a notable reduction in cellular growth and migration, while downregulation of *miR‐152* exerts opposite effects. Further exploration demonstrated that *miR‐152* directly targets *DNMT1*, and ectopic expression of *miR‐152* leads to the downregulation of *DNMT1*. This finding suggests a reciprocal regulatory association between the expression of *miR‐152* and *DNMT1*. Thereby, modulation of *miR‐152/DNMT1* through epigenetic mechanisms significantly influences various processes associated with the malignant behaviour of PCa tumours, particularly in AA PCa patients [102].

### 4.10 Glioma

Glioma, a prevalent malignant neoplasm originating from neuroepithelial tissue within the central nervous system, constitutes 40% to 50% of intracranial tumours. This disease exhibits a poor prognosis and elevated mortality rates. Consequently, the clinical management of glioma necessitates the identification of novel biomarkers for diagnosis, prognosis and therapeutic intervention [103].

#### 4.10.1 MiR‐148/152—DNMT1 Axis

*miR‐148‐3p* is primarily involved in glioblastoma multiforme (GBM) through its direct regulatory effects on *DNMT1* and recombinant human runt‐related transcription factor 3 (*RUNX3*). *MiR‐148‐3p* is significantly reduced in glioma tissues compared to adjacent nontumor tissues and correlated with various factors, including WHO grade, tumour size, prognosis, as well as *DNMT1* and *RUNX3* expressions. This decrease coincided with a *DNMT1* upregulation and *RUNX3* promoter region hypermethylation. Further cellular analysis revealed that the upregulation of *miR‐148‐3p* resulted in apoptosis and cell cycle arrest in U251 and U87 and influenced cell migration. Additionally, overexpression of *miR‐148‐3p* inhibited *DNMT1* expression and *RUNX3* promoter methylation, ultimately leading to *RUNX3* overexpression. Mechanistic analysis discovered a direct interaction between miR‐148‐3p and the 3′‐UTR of *DNMT1*. Subsequently, *miR‐148‐3p* upregulation or *DNMT1* silencing increased *E‐cadherin* expression and decreased *MMP*‐*9*, *MMP‐2*, *N‐cadherin* and *vimentin* expressions. Therefore, *miR‐148‐3p* exhibited direct repression of *DNMT1* expression, inhibiting proliferation and migration in GBM. This regulatory effect is mediated by modulation of the *DNMT1‐RUNX3* axis and the EMT process [104]. Another mechanistic approach by which *DNMT1* may contribute to glioblastoma pathogenesis is the methylation of the *miR‐152* promoter. In this vein, the *miR‐152* promoter region exhibits hypermethylation, the underlying cause of *miR‐152* downregulation in both glioma tissue samples and cell lines. So, *DNMT1* silencing triggers *miR‐152* upregulation, and there is a negative correlation between *miR‐152* expression and the presence of *DNMT* in glioma cell lines. Also, overexpression of *miR‐152* provoked apoptosis in glioma cells, while *miR‐152* suppression facilitated cell proliferation. Ultimately, *miR‐152* exerts regulatory control over the expression of *Runx2*, and the upregulation of *Runx2* nullified the impacts induced by *miR‐152* upregulation. In this manner, the cellular processes of glioma, specifically cell proliferation and apoptosis, are under the regulatory influence of *miR‐152* in conjunction with *Runx2*, and *DNMT1* is critically involved in *miR‐152* hypermethylation and downregulation [105].

#### 4.10.2 LncRNA NEAT1—DNMT1 Axis

*NEAT1*, a lincRNA extensively investigated in the domain of cancer pathologies, is synthesised from the multiple endocrine neoplasia (MEN) site located on chromosome 11q13.1 [106]. *NEAT1* mediates the regulation of *DNMT1* expression through its function as a molecular sponge for *miR‐185‐5p*. *NEAT1* is significantly upregulated, while *miR‐185‐5p* is downregulated in both glioma tissues and cells. Further in vivo and in vitro investigations substantiated the role of *NEAT1* as a ceRNA in facilitating the expression of *DNMT1* and activating mTOR signalling. Notably, *NEAT1* silencing impeded tumour growth and decreased expression levels of Ki‐67, *DNMT1* and mTOR, whereas it concurrently increased *miR‐185‐5p* expression in an in vivo setting. Moreover, *NEAT1* stimulated glioma activity using modulating mTOR signalling, demonstrated in both in vivo and in vitro settings. In this manner, *NEAT1* functioned as an inhibitory factor on mTOR, thereby facilitating glioma tumorigenesis through the *miR‐185‐5p/DNMT1/mTOR* signalling pathway [107].

#### 4.10.3 LncRNA ADAMTS9‐AS2—DNMT1 Axis

*ADAMTS9‐AS2* significantly reduces within tumour tissues compared to normal tissues, with a concomitant inverse correlation between *ADAMTS9‐AS2* expression and tumour grade and prognosis. Elevated levels of *ADAMTS9‐AS2* effectively suppress cell migration in glioma, while *ADAMTS9‐AS2* silencing had the opposite effect. In addition, there is a negative association between the expression of *ADAMTS9‐AS2* and *DNMT1*. Moreover, *DNMT1* silencing resulted in a noteworthy increase in the expression of *ADAMTS9‐AS2*. Therefore, *ADAMTS9‐AS2* functions as a novel tumour suppressor in glioma, and *DNMT1* plays a regulatory role in modulating *ADAMTS9‐AS2* [108].

#### 4.10.4 LncRNA LINC00467—DNMT1 Axis in Glioma

*LINC00467*, a long intergenic non‐coding RNA (lincRNAs) with oncogenic properties, exhibits elevated expression in various malignancies, and its increased levels are frequently associated with unfavourable clinicopathological characteristics [109]. *LINC00467* is significantly upregulated in glioma cells compared to normal tissues, and its overexpression enhances proliferative and invasive capabilities and expedites the cell cycle progression in the G0/G1 phase of U87 and LN229 cells. Importantly, *LINC00467* interacts with *DNMT1* and inhibits the expression of *p53*. Furthermore, the upregulation of *p53* counteracts, to some extent, the heightened impact of *LINC00467* on the proliferative and invasive capabilities of glioma cells. Consequently, *LINC00467* overexpression could stimulate glioma cells' proliferative and invasive abilities by inhibiting *p53* expression by binding *DNMT1* [110].

#### 4.10.5 LncRNA MEG3—DNMT1 in Glioma

The lncRNA known as maternally expressed gene 3 (*MEG3*) is within the imprinted DLK1‐MEG3 locus located on the 14q32.3 region of the human chromosome. Its expression frequently decreases in various types of human tumours and cell lines [111]. Glioma tissues exhibit downregulated expression of *MEG3* due to hypermethylation of its genomic region. In line with previous evidence, administration of 5‐Aza‐2′‐deoxycytidine (5‐AzadC), a DNA methylation inhibitor, resulted in a reduction of abnormal hypermethylation of the *MEG3* promoter and effectively prevented the loss of *MEG3* expression in glioma cells. Therefore, an association exists between *DNMT1* and *MEG3* promoter methylation, wherein *DNMT1* showcased an inverse relationship with *MEG3* expression in gliomas. Additionally, *DNMT1* silencing hindered glioma cells' clone formation and proliferation while concurrently inducing apoptosis. Notably, *DNMT1* silencing contributed to activating *p53* pathways in glioma cells. In this manner, hypermethylation of *MEG3*, facilitated by *DNMT1*, leads to the downregulation of *MEG3* expression and subsequent suppression of p53 signalling pathways in gliomas. Thus, the *MEG3/DNMT1* axis significantly contributes to human glioma pathogenesis, highlighting its potential as a novel therapeutic target in glioma treatment [112].

### 4.11 Lung Cancer

Lung cancer, an extensively recognised malignant neoplasm affecting the respiratory system, has inflicted substantial harm upon human well‐being during the 21st century. The mortality and incidence rates in developing and developed countries are influenced by diverse risk factors, the efficacy of diagnostic techniques and/or the availability of treatment options. Hence, to expedite the development of efficacious clinical interventions targeting lung cancer, the potential molecular mechanism of lung cancer development must be further explored [113].

#### 4.11.1 MiR‐200—DNMT1 Axis

It was discovered that the expression of *miR‐200a* in LUAD cells was significantly reduced and that the direct targeting of 3′‐UTR *GOLM1* resulted in the repression of *GOLM1* expression. Furthermore, when *miR‐200a* was increased, a noticeable inhibition of cell proliferation was observed, effectively impeding the proliferation of LUAD cells induced by *GOLM1* upregulation. It was also discovered that *DNMT1* could downregulate *miR‐200* expression levels, and its excessive expression hindered the suppressive effects of miR‐200a on cellular proliferation. Subsequently, *DNMT1* silencing decreased LAD cell proliferation, which could reverse the introduction of *GOLM1* upregulation. In this manner, the *GOLM1/miR‐200a/DNMT1* axis modulates LUAD cell proliferation [114].

#### 4.11.2 LncRNA HAGLR—DNMT1 Axis

*LncRNA HAGLR*, originating from the HOXD cluster located on the second human chromosome, exhibits increased expression levels across various cancer types [115]. *HAGLR* (also known as HOXD‐AS1) significantly reduces in LUAD tissues. *HAGLR* can diminish LUAD cell proliferation both in vivo and in vitro. Functional investigation disclosed that *HAGLR* exhibits a physical interaction with *DNMT1* and facilitates the recruitment of *DNMT1* on the *E2F1* promoter, thus increasing local DNA methylation. Overall, *HAGLR* facilitated the advancement of LUAD by recruiting *DNMT1* to regulate the methylation patterns and expression of *E2F1* within the promoter region [116].

#### 4.11.3 LncRNA HOXA11‐AS—DNMT1 Axis

Long non‐coding RNA homeobox A11 antisense (*HOXA11‐AS*) increases, while *miR‐148a‐3p* decreases in NSCLC tissues and cells. *HOXA11*‐*AS* silencing hindered the proliferation of NSCLC cells and facilitated cellular apoptosis by directly enhancing the expression of *miR‐148a‐3p*. Furthermore, upregulation of *miR‐148a‐3p* inhibited NSCLC cell proliferation and induced apoptosis. Additionally, *HOXA11‐AS* acted as a ceRNA for *miR‐148a‐3p*, resulting in increased *DNMT1* expression within NSCLC cells. Notably, *DNMT1* overexpression attenuated the impact of *HOXA11‐AS1* depletion on the proliferation and apoptosis of NSCLC cells. In this manner, *HOXA11*‐*AS* contributes to NSCLC tumourigenesis by modulating the *miR‐148a‐3p/DNMT1* axis [117].

### 4.12 Osteosarcoma

Osteosarcoma (OS), a prevalent bone tumour that impacts adolescents and children, necessitates timely identification for successful therapeutic intervention. Hence, there is an imminent need to identify diagnostically and prognostically significant biomarkers, particularly circulating or cellular/tissue biomarkers [118].

#### 4.12.1 MiR‐139‐5p—DNMT1 Axis

In humans, *miRNA‐139*, situated within the genomic locus 11q13.4, exhibits notable antimetastatic and anti‐oncogenic properties [119]. *MiR‐139‐5p* significantly reduces in OS tissues and cell lines. Upregulation of *miR‐139‐5p* effectively inhibits OS growth and migration, while downregulation of miR‐139‐5p induces opposite effects on OS cells. *DNMT1* serves as a specific target of *miR*‐139‐5p. Furthermore, in vivo, findings indicated that the overexpression of *miR‐139‐5p* has a mitigating impact on tumour growth by downregulating the expression of DNMT1. Consequently, *miR‐139‐5p* played a suppressive role in the progression of OS by targeting *DNMT1*, thereby offering novel insights into the underlying molecular mechanism involved in OS development [120].

#### 4.12.2 LncRNA SNHG7—DNMT1 Axis

The expression level of *SNHG7* was significantly elevated in OS tissues compared to adjacent non‐cancerous tissues. *SNHG7* silencing in U2OS and HOS osteosarcoma cell lines led to enhanced cell proliferation, cell cycle arrest at the G0/G1 phase and induction of apoptosis. Mechanistically, *SNHG7* suppressed the transcription of *p53* by forming a complex with *DNMT1*. Subsequently, *p53* upregulation in U2OS cells partially reversed the *SNHG7*‐mediated enhanced cellular proliferation and apoptosis. In this manner, upregulation of *SNHG7* triggers the proliferation of OS cells while concurrently suppressing apoptosis through the modulation of *p53* expression via direct interaction with *DNMT1* [121].

#### 4.12.3 LncRNA NEAT1—DNMT1 Axis

Li et al. explored the role of the *NEAT1/DNMT1* axis in the metastasis of OS. Their findings revealed a substantial upregulation of *NEAT1* expression in both OS tissues and cell lines. Furthermore, they observed a direct association between the *NEAT1* upregulation in OS tissues and unfavourable clinical parameters such as advanced disease stage, poorer prognosis and distant metastasis. Their loss‐ and gain‐of‐function assays disclosed that *NEAT1* positively influenced metastasis in vivo and in vitro. Furthermore, they observed that *NEAT1* ectopic expression led to the induction of EMT. Their mechanistic studies unveiled that *NEAT1* epigenetically silenced *E‐cadherin* expression via its interaction with the *G9a‐DNMT1‐Snail* complex. Their research brings to light a pivotal epigenetic mechanism underlying *NEAT1/DNMT1*‐mediated metastasis [122].

### 4.13 Bladder Cancer

Bladder cancer (BCa) represents the second most prevalent urological malignancy. The majority (approximately 75%) of recently identified instances pertain to non‐muscle‐invasive bladder cancer (NMIBC), with the remaining diagnoses attributed to muscle‐invasive bladder cancer (MIBC). Notably, NMIBC patients experience a notable propensity for relapse and advancement. Consequently, an imperative demand exists for dependable prognostic biomarkers to enhance comprehension of disease occurrence and progression [123].

#### 4.13.1 MiR‐152‐3p—DNMT1 Axis

Liu et al. indicated a substantial increase in DNMT1 expression in both BCa tissues and cells. Additionally, when *DNMT1* expression was silenced, it resulted in the inhibition of tumour growth in vivo. They unveiled that *miR‐152‐3p* exerted an inhibitory effect on *DNMT1*, and the *DNMT1* upregulation reinstated the cellular functionality of *miR‐152‐3p* in BCa cells. Furthermore, *DNMT1* modulated the expression of *PTEN* by influencing DNA methylation in its promoter region. Thus, their investigation effectively validated the involvement of *DNMT1*‐mediated DNA methylation while elucidating a novel regulatory pathway involving *miR‐152/DNMT1/PTEN* in BCa. Consequently, these findings offer potential prospects for diagnostic and therapeutic targets in BCa [124].

#### 4.13.2 MiR‐148a—DNMT1 Axis

*miR‐148a* exhibits a reduction in urothelial carcinoma of the bladder (UCCB) cell lines, and its upregulation resulted in a decline in cell viability attributed to enhanced apoptosis rather than a suppression of proliferation. Furthermore, *miR‐148a* partially modulates this impact by downregulating the expression of *DNMT1*. Notably, combined treatment of *miR‐148a* and either cisplatin or doxorubicin in cells exhibits an additive or synergistic effect on inducing apoptosis. In this manner, *miR‐148a* exhibits tumour‐suppressive properties in UCCB, and the *miR‐148a/DNMT1* axis holds promising potential as an innovative therapeutic approach for addressing this particular malignancy [125].

#### 4.13.3 MiR‐424—DNMT1 Axis in BCa

*miR‐424* significantly upregulated upon inhibition of *DNMT1* in BCa cells. Additionally, an inverse correlation exists between *miR‐424* staining and the immunoreactivity of *DNMT1*, providing evidence for the pivotal involvement of *DNMT1* in suppressing *miR‐424* expression. Notably, elevated levels of *miR‐424* suppressed the tumour growth rate and invasive potential, as examined both in vitro and in vivo. Furthermore, the EGFR pathway transmits the *miR‐424* signal, which governs cellular growth and EMT. Consequently, these findings emphasise the prospective significance of the *miR‐424/DNMT1* axis as a molecular prognosticator and therapeutic target in the context of BCa [126].

#### 4.13.4 LncRNA DBCCR1‐003—DNMT1 Axis

Recent experimental investigation revealed downregulation of *DBCCR1‐003* and *DBCCR1*, alongside an upregulation of *DNMT1* and *DBCCR1* gene promoter hypermethylation in both BCa tissues and the T24 cell line. Furthermore, silencing *DNMT* via 5‐aza‐2‐deoxycytidine (DAC) or enhanced expression of *DBCCR1‐003* resulted in *DBCCR1* overexpression within T24 cells, achieved through the reversal of promoter hypermethylation and the disruption of *DNMT1* binding to the *DBCCR1* promoter. Notably, a physical association exists between *DBCCR1*‐*003* and *DNMT1*. Moreover, it was observed that the binding between these two molecules increased when the methylation of the *DBCCR1* promoter was inhibited. These findings suggest that *DBCCR1*‐*003* has the potential to bind with *DNMT1* and impede the *DNMT1*‐mediated methylation process of *DBCCR1* [127].

### 4.14 Oesophageal Cancer

Oesophageal cancer is the predominant malignancy in the gastrointestinal (GI) system and is characterised by suboptimal prognosis and survival rates. Over recent decades, numerous endeavours have been undertaken to identify efficacious therapeutic strategies; however, these approaches have encountered various challenges. Therefore, the identification of novel molecular biomarkers plays a pivotal role in the exploration of alternative therapeutic strategies for the management of these malignancies [128].

#### 4.14.1 MiR‐148a‐3p—DNMT1 Axis

*miR‐148a‐3p* directly targets *DNMT1*, and a negative correlation exists between the expression levels of *miR‐148a‐3p* and *DNMT1* in the context of oesophageal cancer. Excessive expression of *miR‐148a‐3p* in oesophageal cancer cells triggers inhibition of proliferation and invasion, as well as an enhancement of apoptosis. This suggests that *miR‐148a‐3p* potentially governs cell proliferation and invasion in oesophageal cancer by selectively targeting *DNMT1*. Hence, the *miR‐148a‐3p*/*DNMT1* axis holds promise as a prospective therapeutic target for future interventions [129].

#### 4.14.2 MiR‐124‐3p—DNMT1 Axis

*MiR‐124* is abundantly present as a miRNA in the brain, yet its expression is observed across diverse human and animal tissues, contributing to various disorders, including cancer [130]. The expression levels of *miR‐124‐3p* significantly decrease in oesophageal squamous cell carcinoma (ESCC) tissues, demonstrating a strong association with the increased proliferation and migration capabilities of ESCC. In ESCC tissues and cell lines, direct targeting of the mRNA 3′UTR region of *BCAT1* by *miR‐124‐3p* was detected. Furthermore, a regulatory pathway governs the expression of *miR‐124‐3p* in ESCC, explicitly implicating the involvement of *DNMT1*‐mediated hypermethylation‐induced silencing. Notably, *DNMT1* displayed augmented expression levels within ESCC tissues and cell lines. Consequently, the *DNMT1/miR‐124/BCAT1* axis governs the advancement and advancement of ESCC [131].

#### 4.14.3 MiR‐126—DNMT1 Axis

*MiR*‐*126*, situated in the 7th intron of *EGFL7* on human chromosome 9, has been implicated in the pathogenesis of GI malignancies [132]. In ESCC, *miR‐126* exhibited substantial downregulation, and diminished expression of *miR‐126* was attributed to promoter hypermethylation impacting its host gene, *Egfl7*. Also, *DNMT1* abnormally increased in ESCC, which led to the excessive methylation of *Egfl7*. Interestingly, *miR‐126* upregulation resulted in the suppression of *DNMT1*, suggesting the presence of a regulatory feedback loop. Also, it was found that *miR‐126* directly targets *ADAM9*. Furthermore, *miR‐126* ectopic expression or repression of *ADAM9* resulted in diminished cellular proliferation and migration in ESCC, accomplished through the restraint of epidermal growth factor receptor‐AKT signalling. In this manner, *miR‐126* holds promise as a prognostic marker for ESCC and proposes the involvement of a novel ‘*DNMT1*‐*miR‐126* epigenetic circuit’ in the progression of ESCC [133].

#### 4.14.4 LncRNA LUCAT1—DNMT1 Axis

*LUCAT1* expression displays upregulation in ESCC cell lines and cancerous tissue relative to normal cells and adjacent non‐malignant tissues. Silencing LUCAT1 in KYSE‐30 cells by decreasing DNA methylation exhibited a decrease in cellular proliferation, initiation of apoptosis and upregulation of tumour‐suppressor genes. Furthermore, the knockdown of *LUCAT1* resulted in a decline in DNMT protein levels, while transcription remained unaffected. Mechanistic investigation disclosed that *LUCAT1* plays a role in modulating the stability of *DNMT1*, leading to the inhibition of tumour suppressor gene expression via DNA methylation. Consequently, this molecular mechanism contributes to the initiation and metastasis of ESCC. In this manner, *LUCAT1/DNMT1* is a prospective candidate for pharmaceutical advancement and a discerning indicator for ESCC [134].

### 4.15 Cervical Cancer

The high prevalence and fatality rate of CC, a condition specific to females, has prompted scientists to contemplate innovative approaches and formulate novel treatment protocols and strategies [135].

#### 4.15.1 MiR‐148a‐3p—DNMT1 Axis

Chen et al. investigated the involvement of *miR‐148a‐3p* in CC. They revealed a notable reduction in the expression levels of *miR‐148a‐3p* within CC tissues compared to normal cervical tissues. They observed a significant decrease in the growth rate of CC cells upon the increased expression of *miR‐148a‐3p*. Their luciferase reporter assay successfully identified *DNMT1* as the specific target gene regulated by *miR‐148a‐3p*. Also, a negative association exists between the expression levels of the undifferentiated embryonic cell transcription factor‐1 (*UTF1*) and the expression levels of *DNMT1* in CC tissues. Thus, *DNMT1* silencing resulted in an elevation of *UTF1* expression and reduced *UTF1* promoter methylation levels. These findings provide evidence for the regulatory role of *DNMT1* methylation in modulating the expression levels of *UTF1* in CC cells. Collectively, *miR‐148a‐3p* potentially hinders the growth of CC cells by modulating the expression levels of *DNMT1/UTF1*, thereby offering promising therapeutic targets for CC [136].

#### 4.15.2 LncRNA TCF7—DNMT1 Axis

Chen et al. investigated the biological significance of *lncRNA TCF7* in CC. They found that suppressing endogenous HPV‐16 E6 had a significant inhibitory effect on *DNMT1* expression. Furthermore, silencing *DNMT1* triggers a notable augmentation in *miR‐155* expression levels. They elucidated that *miR‐155* directly targets 3′ UTR *TCF‐7*. Additionally, it was disclosed that restraining *TCF‐7* activity resulted in suppressed migratory capabilities of CC cells. Further in vivo investigation disclosed that suppressing *LncRNA TCF7* effectively mitigates the growth rate of CC cells. In this manner, the *miR‐155/DNMT1/TCF‐7* axis exhibits potential regulatory capabilities over the migration processes of CC cells, thereby indicating its significance as a crucial regulator in the development of CC [137].

#### 4.15.3 LncRNA LINP1—DNMT1 Axis

*LINP1* expression increases in CC tissues compared to adjacent normal tissue and healthy human cervical epithelial cell lines (HUCEC). Surprisingly, *LINP1* reduction markedly suppressed the proliferative capacity of CC cells, facilitated apoptosis and substantially impeded the *vivo* growth of CC tumours. Additionally, *LINP1* plays a critical role in the recruitment of *DNMT1*, *LSD1* and *EZH2* by *LINP1*, consequently leading to diminished expression levels of *PRSS8* and *KLF2*. So, the downregulation of *LINP1* resulted in both *KLF2* and *PRSS8* overexpression within CC cells. Further, increased expression of *PRSS8* and *KLF2* restrains cellular proliferation while promoting cellular apoptosis in CC. Additionally, inhibition of *KLF2* and *PRSS8* counteracted the suppressive effects on cell proliferation induced by the silencing of *LINP1*. Therefore, *LINP1* promotes CC progression by recruiting *DNMT1* and inhibiting *KLF2* and *PRSS8*. In this manner, targeting *LINP1* may hold significant potential as a therapeutic approach for treating CC [138].

### 4.16 Head and Neck Squamous Cell Carcinoma

Head and neck squamous cell carcinoma (HNSCC) is the prevailing histological neoplasm originating within the head–neck region. Despite the implementation of surgical interventions, radiotherapy and chemotherapy for advanced stages (3 and 4), the 5‐year survival rate remains notably low. Consequently, a pressing imperative exists to innovate novel diagnostic methodologies and targeted therapeutic approaches [139].

#### 4.16.1 MiR‐142‐3p—DNMT1 Axis

Li et al. explored the involvement of the *DNMT1/miR‐142‐3p/ZEB2* axis in nasopharyngeal carcinoma (NPC). Their in silico findings initially identified *miR‐142‐3p* as the most strongly associated with distant‐metastasis‐free survival and exhibiting downregulation in paraffin‐embedded NPC samples displaying distant metastasis. Additionally, the *miR‐142* locus exhibits hypermethylation in metastatic NPC and is correlated with the *miR‐142‐3p* reduction. Further, the silencing of *miR‐142‐3p* through epigenetic mechanisms involving *EZH2*‐mediated recruitment of *DNMT1* resulted in the suppression of NPC cell metastasis. *ZEB2* serves as a specific and functionally significant target of *miR‐142‐3p* in NPC. Consequently, *miR‐142‐3p* functions as a crucial suppressive modulator in the metastasis of NPC while also uncovering a *DNMT1*‐mediated epigenetic mechanism responsible for *miR‐142‐3p* inhibition [140].

#### 4.16.2 MiR‐148‐3p—DNMT1 Axis

Jili et al. focused on exploring the biological significance of the *DNMT1/miR‐148‐3p/RUNX3* axis in Laryngeal squamous cell carcinoma (LSCC). They observed a significant reduction and increased methylation of the *RUNX3* gene in LSCC compared to the corresponding normal tissue. They further observed that the RUNX3‐enforced expression suppressed LSCC cell migration and proliferation, while *RUNX3* suppression had the opposite effect. They discovered a regulatory relationship between *miR‐148a‐3p* and *RUNX3*, where they observed a significant reduction in the expression level of miR‐148a‐3p, which was positively associated with the expression of *RUNX3* in LSCC. They additionally determined that *miR‐148a‐3p* selectively targeted *DNMT1* within the context of LSCC. They subsequently revealed that *DNMT1* suppression resulted in *RUNX3* overexpression while concurrently impeding the migratory and proliferative capacities of LSCC cells. In summary, *miR‐148a‐3p* can influence the expression of *RUNX3* by altering *DNMT1*‐mediated DNA methylation in LSCC [141].

### 4.17 Melanoma

Melanoma, the most lethal variant of skin cancer, presents ongoing hurdles in its management, encompassing the need for precise prognostication of individuals amenable to adjuvant therapies and the timely identification of relapses. The difficulties have stimulated inquiry into biomarkers that hold the potential to serve as a therapeutic, prognostic and diagnostic aid [142].

#### 4.17.1 MiR‐211—DNMT1 Axis

Yu et al. examined the *DNMT1/miR‐211/RAB22A* axis in melanoma. They initially validated the expression of *miR‐211* in melanoma cell lines and noted a positive correlation between its reduction and enhanced *DNMT1* expression. Their experimental findings provided substantiation for a negative association between *DNMT1* and *miR‐211* expression and the ability of *DNMT1* to regulate DNA methylation in the *miR‐211* promoter region. Additionally, they identified a direct interaction between *miR‐211* and *RAB22A* while establishing the inhibitory impact of *miR‐211* on *RAB22A* expression. They also observed that *RAB22A* silencing enhanced epithelial characteristics and compromised mesenchymal features in melanoma cells, indicating that *miR‐211* regulates the process of EMT in melanoma cells by negatively regulating *RAB22A*. So *DNMT1*‐mediated promoter methylation functions to suppress miRNA activity within melanoma. Furthermore, *miR‐211* functions as a tumour suppressor in melanoma by negatively regulating *RAB22A*. Therefore, the *DNMT1/miR‐211/RAB22A* axis offers a fresh perspective on the aetiology of melanoma, specifically concerning its involvement in the EMT pathway [143](Table 1).

**TABLE 1:** Major miRNAs and their relationship in human cancer.

miRNAs	Author	Cancer	Expression levels	miRNAs relationship with DNMT1	Biological significance	Ref.
miR‐148/152	Zhao et al.	Hepatocellular	Downregulated	miR‐152 directly target DNMT1	HBX via miR‐152 reduction and upregulating DNMT1 expression, involved in HCC	[39]
	Long et al.	Hepatocellular	Downregulated	miR‐148a Directly target DNMT1	Overexpression of miR‐148a significantly inhibits HCC cell proliferation	[41]
	Shi et al.	Gastric	Downregulated	miR‐148a Directly target DNMT1	miR‐148a significantly reduced cell migratory	[51]
	Yan et al.	Gastric	Downregulated	miR‐148a Directly target DNMT1	miR‐148a significantly reduced cell proliferation	[52]
	Zuo et al.	Gastric	Downregulated	miR‐148a Directly target DNMT1	miR‐148a downregulation led to GC progression	[53]
	Zuo et al.	Gastric	Downregulated	There is a negative feedback regulatory loop between miR‐148a and DNMT1	over‐expression of miR‐148a inhibited cell proliferation	[54]
	Wang et al.	Colorectal	Downregulated	DNMT1 involved in miR‐152‐3p hypermethylation	miR‐152‐3p exert inhibitory effects on the progression of CRC	[68]
	Xie et al.	Pancrease	Downregulated	miR‐152 directly target DNMT1	miR‐152 trigger substantial growth suppression	[144]
	Hong et al.	Pancrease	Downregulated	miR‐148a directly target DNMT1	miR‐148a led to a significant inhibition of cell proliferation, and migration	[74]
	Zhan et al.	Pancrease	Downregulated	There is a negative feedback regulatory loop between miR‐148a and DNMT1	miR‐148a led to a significant inhibition of cell proliferation, and migration	[75]
	Azizi et al.	Pancrease	Downregulated	miR‐148b/152 directly target DNMT1	miR‐148b/152 significantly reduced cell proliferation	[76]
	Wang et al.	AML	Downregulated	There is a negative feedback regulatory loop between miR‐148a and DNMT1	miR‐148a suppressed cellular proliferation while fostering apoptosis	[80]
	Wang et al.	Non‐Hodgkin lymphoma	Downregulated	miR‐152 directly target DNMT1	miR‐152 induce apoptosis and impede cell proliferation.	[81]
	Sengupta et al.	Breast	Downregulated	There is a negative feedback regulatory loop between miR‐152 and DNMT1	miR‐152 impede metastasis	[90]
	Xu et al.	Breast	Downregulated	There is a negative feedback regulatory loop between miR‐148a/152 and DNMT1	miR‐148a/miR‐152 results in significant inhibition of angiogenesis, colony formation and cell proliferation	[91]

	Chen et al.	Endometrial	Downregulated	miR‐148b directly target DNMT1	miR‐148b induce apoptosis and impede cell proliferation.	[98]
	Hsiao et al.	Endometrial	Downregulated	miR‐148a directly target DNMT1	miR‐148a exert inhibitory effects on the progression of Endometriosis	[99]
	Sengupta et al.	Prostate	Downregulated	miR‐148a target DNMT1	miR‐148a induce apoptosis and impede cell proliferation.	[101]
	Theodore et al.	Prostate	Downregulated	There is a negative feedback regulatory loop between miR‐152 and DNMT1	miR‐152 led to a notable reduction in cellular migration	[102]
	Li et al.	Glioma	Downregulated	miR‐148 directly target DNMT1	miR‐148 induce apoptosis and impede cell proliferation	[104]
	Zhang et al.	Glioma	Downregulated	DNMT1 involved in MiR‐152 hypermethylation	miR‐152 induce apoptosis and impede cell proliferation	[105]
	Sun et al.	Glioma	Downregulated	miR‐148 directly target DNMT1	miR‐152 impede cell proliferation, and invasion	[145]
	Liu et al.	Bladder	Downregulated	miR‐152‐3p directly target DNMT1	miR‐152‐3p exerted an inhibitory effect on migration	[124]
	Lombard et al.	Bladder	Downregulated	miR‐148a directly target DNMT1	miR‐148a induce apoptosis and impede cell proliferation	[125]
	Wang et al.	Oesophageal	Downregulated	miR‐148a‐3p directly target DNMT1	miR‐148a induce apoptosis and impede cell proliferation, and metastasis	[129]
	Chen et al.	Cervical	Downregulated	miR‐148a‐3p directly target DNMT1	miR‐148a‐3p led to decreased cell growth	[136]
	Jili et al.	Laryngeal squamous cell	Downregulated	miR‐148a‐3p directly target DNMT1	miR‐148a‐3p impede cell proliferation, and metastasis	[141]
Lu et al.	Nasopharyngeal	Downregulated	There is a negative feedback regulatory loop between miR‐152 and DNMT1	miR‐152 impede metastasis	[146]
*MiR‐185*	Qadir et al.	Hepatocellular	Downregulated	miR‐185 directly target DNMT1	miR‐185 inhibited cell proliferation and invasion	[42]
MiR‐378a‐3p	Zhu et al.	Hepatocellular	Downregulated	DNMT1 involved in MiR‐378a‐3p hypermethylation	miR‐378a‐3p downregulation led to a significant increase in angiogenesis	[44]
MiR‐30b	Qiao et al.	Gastric	Downregulated	DNMT1 involved in MiR‐30b hypermethylation	miR‐30b prevented cell migration	[56]
MiR‐34a	Ma et al.	Pancrease	Downregulated	DNMT1 involved in miR‐34a hypermethylation	miR‐34a downregulation led to pancreatic cancer	[72]
MiR‐377	Azizi et al.	Pancrease	Downregulated	miR‐377 directly target DNMT1	miR‐377 prevented cell proliferation and induce apoptosis	[78]
MiR‐497	Liu et al.	Breast	Downregulated	DNMT1 involved in miR‐497 hypermethylation	miR‐497 suppressed the metastatic potential	[89]
MiR‐142‐5p	Li et al.	Nasopharyngeal	Downregulated	DNMT1 involved in miR‐142‐5p hypermethylation	miR‐142‐3p suppresses metastasis	[140]
MiR‐200	Yang et al.	Lung	Downregulated	DNMT1 involved in miR‐200 hypermethylation	miR‐142‐5p impeded the proliferation	[114]
MiR‐139‐5p	Shi et al.	Osteosarcoma	Downregulated	miR‐139‐5p directly target DNMT1	miR‐139‐5p inhibited cell proliferation, and migration	[120]
MiR‐424	Wu et al.	Bladder	Downregulated	DNMT1 involved in miR‐424 hypermethylation	miR‐424 impeded invasion	[126]
MiR‐124	Zeng et al.	Oesophageal	Downregulated	DNMT1 involved in miR‐124 hypermethylation	miR‐124 inhibited cell proliferation, and migration	[131]
MiR‐211	Yu et al.	Melanoma	Downregulated	DNMT1 involved in miR‐211 hypermethylation	miR‐211 inhibited cell migration	[143]

## 5 LncRNAs/miRNA‐DNMT1 Axis in Cancer Therapy Resistance

Despite considerable advancements in comprehending the genetic factors implicated in cancer pathogenesis, numerous obstacles persist in the realm of cancer therapeutics, posing a formidable threat to the well‐being and mortality rates of individuals who have cancer worldwide. Hence, there is a pressing need to introduce fresh perspectives to understand better the underlying mechanisms that contribute to treatment resistance in tumour therapy. By understanding these mechanisms comprehensively, novel therapeutic approaches can be devised and implemented in the foreseeable future. Recent scientific investigations illustrated the pivotal role of the ncRNA‐*DNMT1* axis in regulating therapy resistance by influencing various biological processes [147]. Therefore, the present review focuses on the ncRNA‐*NMT1* axis in the therapy resistance of tumours.

### 5.1 MiRNAs/DNMT1 Axis in Cisplatin Resistance

Han et al. explored the involvement of the *miR‐30* family in the resistance of OC cells to cisplatin. Within their study, they observed that *miR‐30c‐5p* and *miR‐30a‐5p* exhibited a substantial reduction in cisplatin‐resistant CP70 cells. This decrease was attributed to the induction of aberrant methylation caused by increased *DNMT1*. They additionally observed that *miR‐30a/c‐5p* exerted direct inhibitory effects on Snail and *DNMT1*. Further, *miR‐30a/c‐5p* enforced expression or the suppression of *DNMT1* and Snail enhanced sensitivity to cisplatin and a partial reversal of EMT in CP70 cells. In contrast, *miR‐30a/c‐5p* suppression or the *DNMT1* and Snail ectopic expression triggered cisplatin resistance and partial EMT in cisplatin‐sensitive A2780 cells. Notably, a reciprocal relationship exists between *miR‐30a/c‐5p* and *DNMT1*, a robust indicator of cisplatin resistance and EMT in ovarian cancer. This finding highlights a prospective target for enhancing anti‐cancer therapy [148]. In addition, Xiang et al. observed a notable downregulation of two specific microRNAs, namely *miR‐185* and *miR‐152*, in cisplatin‐resistant ovarian cell lines A2780/DDP and SKOV3/DDP, when compared to their respective sensitive parent lines A2780 and SKOV3. They revealed that upregulation of *miR‐152* or miR‐185 resulted in heightened sensitivity to cisplatin in A2780/DDP and SKOV3/DDP cells by impeding cell proliferation and facilitating apoptosis. Subsequently, they validated that these particular microRNAs exerted their effects by directly suppressing *DNMT1*. Importantly, administration of SKOV3/DDP cells with *miR‐152* mimics via intraperitoneal injection in CD‐1/CD‐1 nude mice resulted in an observed enhancement of cisplatin sensitivity within an in vivo context. Furthermore, their survival assays in A549 and HepG2 cells indicated that the microRNAs implicated in cisplatin sensitivity exhibited cell type‐specific associations. So, the *miR‐152*/*miR‐185*/*DNMT1* axis is involved in both in vitro and in vivo cisplatin resistance in OC [149]. Moreover, Sui et al. explored the biological significance of *miR‐148b* in the progression of chemoresistance within lung cancer. Their findings exhibited a decrease in the *miR‐148b* expression and an increase in *DNMTs* expression in cisplatin‐resistant human NSCLC cell lines, namely SPC‐A1/DDP and A549/DDP, in comparison to their parental counterparts SPC‐A1 and A549. Overexpression of *miR‐148b* resulted in a reduction in *DNMT1* expression, enhanced cellular sensitivity to cisplatin and promoted cisplatin‐induced apoptosis in SPC‐A1/DDP and A549/DDP cells. In addition, silencing *miR‐148b* resulted in *DNMT1* upregulation, alongside a reduction in cell sensitivity to cisplatin treatment in SPC‐A1 and A549 cells. They subsequently revealed that *miR‐148b* suppresses *DNMT1* expression by targeting the 3'UTR in A549 and A549/DDP cell lines. Notably, *DNMT1* silencing enhances the susceptibility of A549/DDP cells to cisplatin, while *DNMT1* upregulation counteracts the pro‐apoptotic impact induced by the introduction of *miR‐148b* mimic. Consequently, *miR‐148b* effectively counteracts the resistance to cisplatin in non‐small cell cancer cells by negatively modulating the expression of *DNMT1* [150].

### 5.2 MiRNAs/DNMT1 Axis in Doxorubicin Resistance

Congras et al. explored the involvement of *miR‐125b* in the progression of doxorubicin resistance in nucleophosmin‐anaplastic lymphoma kinase (NPM‐ALK) (+) anaplastic large‐cell lymphoma (ALCL). Their findings indicate that the expression of miR‐125b is reduced in NPM‐ALK (+) cell lines and samples obtained from patients, primarily due to hypermethylation occurring within its promoter region. In their investigation, they observed that the activity of NPM‐ALK, in conjunction with DNA topoisomerase II (Topo II) and *DNMT1*, plays a pivotal role in *miR‐125b* silencing through DNA hypermethylation. Interestingly, they found that *miR‐125b* silencing could be effectively counteracted by inhibiting DNMTs using decitabine or by obstructing DNA Topo II's function using doxorubicin or etoposide. Additionally, they revealed that doxorubicin administration in NPM‐ALK (+) cell lines resulted in elevated levels of *miR‐125b* through the inhibition of the *DNMT1* binding to the *MIR125B1* promoter and the subsequent downregulation of *BAK1*, a target gene associated with pro‐apoptotic activities. They subsequently revealed that reversing *miR‐125b* suppression, enhancing *miR‐125b* concentrations and diminishing *BAK1* expression exhibited a correlation with the reduced effectiveness of doxorubicin, implying the presence of a pharmacoresistance mechanism. The *DNMT1/miR‐125b* pathway holds potential as a biomarker for resistance in cases of ALK (+) ALCL [151].

### 5.3 MiRNAs/DNMT1 Axis in Temozolomide Resistance

**FIGURE 3:**
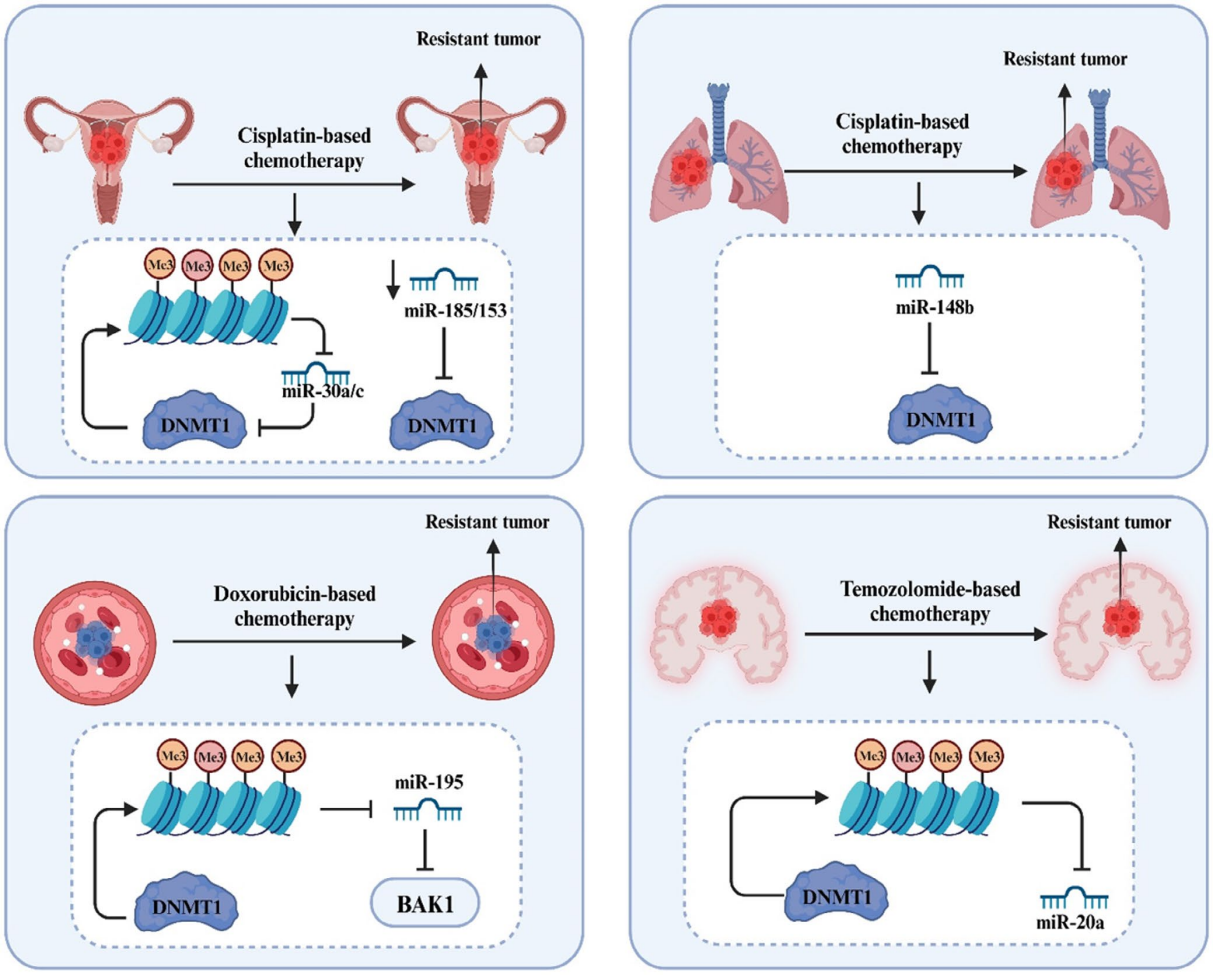
A schematic representation of miRNAs/DNMT1 axis in cancer therapy resistance.

Zhou et al. explored the potential correlation between the *DNMT1/miR‐20a* axis and the sensitivity of glioma cells to temozolomide (TMZ). They revealed that the expression of *DNMT1* was observed to be decreased, the methylation of the *miR‐20a* promoter was attenuated, and the levels of *miR‐20a* were elevated in TMZ‐resistant U251 cells compared to the parental U251 cells. It was observed that the reduction of TMZ sensitivity in U251 cells occurred due to methyltransferase silencing through treatment with 5‐aza‐2′‐deoxycytidine Additionally, they noted that in U251/TM cells, there existed an inverse relationship between *DNMT1* expression and *miR‐20a* expression, while a positive correlation was found between *DNMT1* expression and both TMZ sensitivity and leucine‐rich repeats and immunoglobulin‐like domains 1 expression. These effects were subsequently reversed upon alterations in *miR‐20a* expression. In their study, *DNMT1* upregulation increased apoptotic events in U251/TM cells, which was counteracted by *miR‐20a* mimic. Conversely, the *DNMT1* suppression mitigated U251/TM cell apoptosis, and this effect was nullified upon treatment with a *miR‐20a* inhibitor. They finally disclosed that pretreatment with pcDNA‐DNMT1 suppressed the growth of U251/TM xenograft tumours, while pretreatment with *DNMT1*‐small hairpin RNA enhanced their growth. In summary, *DNMT1* facilitated chemosensitivity by attenuating methylation levels in the promoter region of *miR‐20a* within glioma cells [152](Figure 3).

## 6 LncRNAs/miRNA‐DNMT1 Axis in Cancer Stem Cell

Stem cells possess two pivotal characteristics, specifically the capacity for self‐renewal and the potential to undergo differentiation into various cell lineages endowed with distinct functional roles. These inherent attributes are also exhibited by cancer stem cells (CSCs). These cells have been identified in various cancer types, contributing to tumour formation. A recent study revealed that the axis involving ncRNA and *DNMT1* plays a crucial role in regulating the activity of CSCs. Therefore, in the following section, we explain the effects of the ncRNA‐*DNMT1* axis on CSC activity and their impact on tumorigenesis [153].

### 6.1 MiR‐34a/DNMT1 Axis

LCSCs displayed increased *DNMT1* activity and expression, reduced *miR‐34a* expression accompanied by enhanced promoter methylation, and heightened stemness properties compared to the original liver cancer cells. Also, *DNMT1* silencing resulted in the repression of *DNMT1* itself, accompanied by an increase in *miR‐34a* levels through demethylation of its promoter region. This inhibition also led to a reduction in stemness characteristics within LCSCs. Furthermore, overexpression of *miR‐34a* resulted in the repression of stemness properties, while silencing *miR‐34a* exert opposite effects. Furthermore, overexpression of *miR‐34a* successfully mitigated the impact of elevated *DNMT1* levels on the stem cell characteristics of LCSCs while leaving *DNMT1* expression unaffected. Ultimately, *FOXM1* serve as a direct target of miR‐34a within LCSCs. Therefore, *DNMT1's* abnormal activity results in promoter methylation and subsequent repression of miR‐34a, thereby leading to *FoxM1* overexpression through the promotion of LCSC stemness. Thereby, inhibition of *DNMT1/miR‐34a*‐mediated *FOXM1* overexpression could potentially suppress liver cancer by selectively targeting LCSCs [154]. Also, *DNMT1/miR‐34a* axis plays a crucial role in regulating osteosarcoma cancer stem‐like cells (OSLCs). In this regard, higher *DNMT1* levels, primarily through the induction of methylation in the miR‐34a promoter, significantly reduce its expression and are associated with increased stemness of OSLCs. Moreover, silencing *DNMT1* is associated with demethylation of the *miR‐34a* promoter and upregulation of miR‐34a expression, which leads to the suppression of stemness in OSLCs in a dose‐dependent manner. Thereby, abnormal activation of *DNMT1* induces promoter methylation of *miR‐34a*, resulting in its downregulation, thereby enhancing and maintaining the stemness characteristics of OSLCs [155].

### 6.2 MiR‐497‐5p/DNMT1 Axis

According to recent experimentation, high *SALL4* expression is associated with lower progression‐free survival (PFS) rates, and *SALL4* inhibition led to diminished capabilities of colony formation, proliferation, drug resistance and migration in vitro. Furthermore, there is a direct and inverse relationship between *miR‐497‐5p* and *SALL4*. Moreover, suppression of *miR‐497‐5p* led to the enhancement of stem‐like properties in choriocarcinoma CSLCs. In addition, increased expression of *SALL4* and *miR‐497‐5p* reduction facilitates the progression of choriocarcinoma within an in vivo. Notably, *DNMT1*/*3B* overexpression, facilitated by the upregulation of *SALL4*, hindered the expression of miR‐497‐5p by promoting hypermethylation. Thus, the *miR‐497‐5p/SALL4/DNMT1/3B* axis emerged as a critical factor in fostering the stemness phenotype of choriocarcinoma [156].

### 6.3 MiR‐17‐92 Cluster/DNMT1 Axis

Pancreatic CSCs, irrespective of their heterogeneity or polyclonality within the analysed tumours, exhibit elevated levels of *DNMT1* activity and DNA methylation. Moreover, applying pharmacological or genetic methods to target *DNMT1* in CSCs specifically decreased their self‐renewal and in vivo tumour formation capacity. These findings establish *DNMT1* as a promising therapeutic target for CSCs. Further, the *miR‐17‐92* cluster, which consists of six individual members (*miR‐17*, *18a*, *19a*, *19b*, *20a* and *92a*), exhibited hypermethylation in CSCs compared to non‐CSCs. Additionally, *miR‐17‐92* upregulation decreased CSC self‐renewal potential, in vivo tumour formation ability, and resistance to chemotherapy. Furthermore, suppression of the *miR‐17‐92* cluster in differentiated cells resulted in a contrasting outcome, inducing non‐CSCs to exhibit characteristics resembling CSCs. In this manner, *DNMT1* primarily functions by repressing the *miR‐17‐92* cluster, significantly influencing PDAC CSCs maintenance. These results highlight the *DNMT1*/*miR‐17‐92* cluster axis as a critical regulator of biological processes in CSCs and offer a compelling basis for developing epigenetic modifiers to target CSC plasticity [157].

### 6.4 MiR‐137/DNMT1 Axis

There is a notable upregulation of *BCL11A* in TNBC, while the expression of *miR*‐*137* is significantly decreased in both TNBC tissues and cell lines. The expression of *BCL11A* is downregulated at both the mRNA and protein levels by *miR‐137* through direct targeting of its 3′ UTR. Additionally, upregulation of *miR‐137* or silencing of *BCL11A* resulted in a decrease in the number of tumorspheres and the proportion of CSCs in both MDA‐MB‐231 and SUM149 cell lines, while also exerting an inhibitory effect on tumour growth in vivo. Additionally, an interaction exists between *BCL11A* and *DNMT1* within TNBC cells. Notably, the inhibition of either *DNMT1* or *BCL11A* results in a compromised capacity for cancer stemness and tumorigenesis in TNBC, which is achieved through the suppression of ISL1 expression both in vivo and in vitro. Furthermore, *miR‐137* disrupts the interaction between *BCL11A* and *DNMT1*, reducing cancer stemness and inhibiting tumour progression in TNBC [158].

### 6.5 MiR‐126/DNMT1 Axis

**FIGURE 4:**
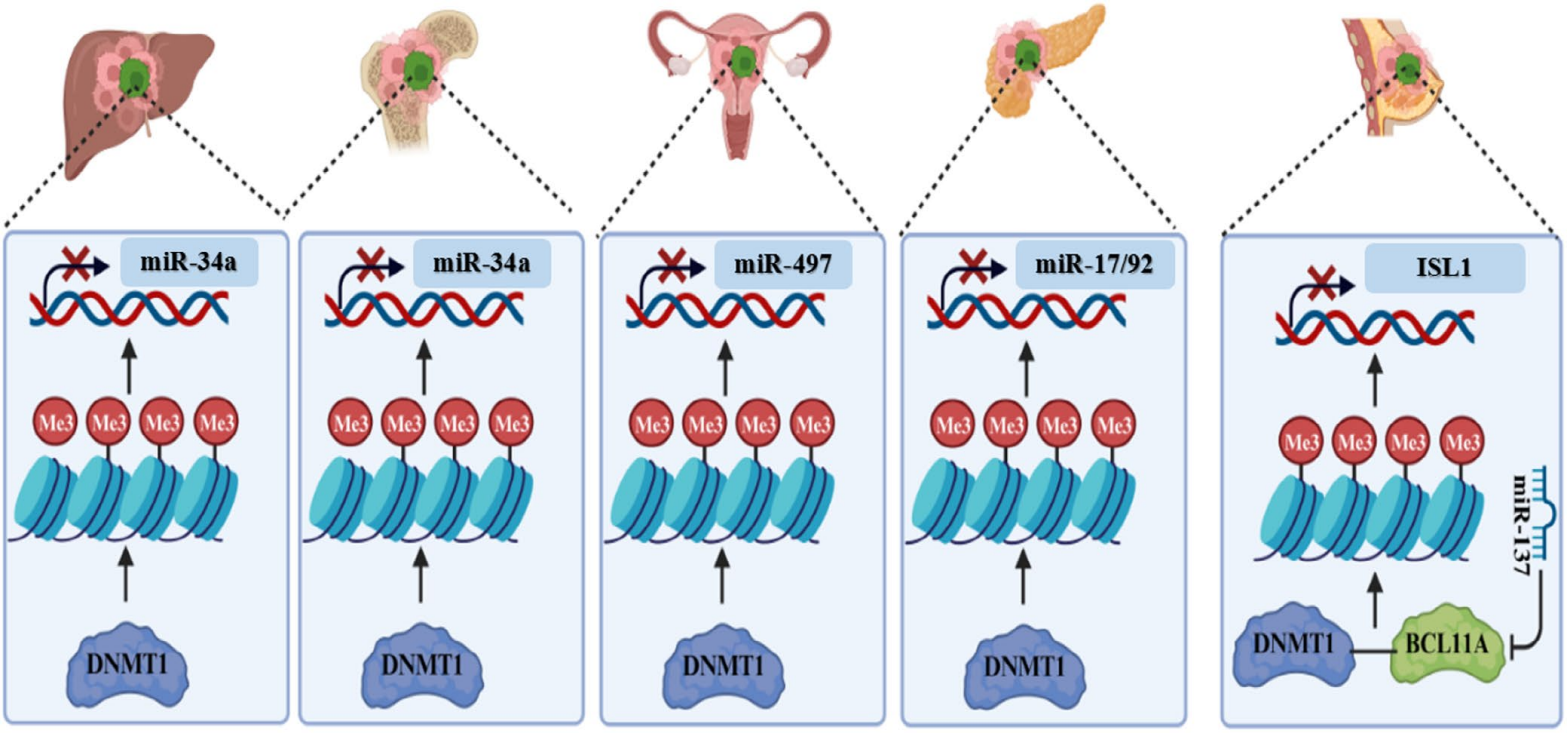
A schematic representation of miRNAs/DNMT1 axis in cancer stem cells.

Ding et al. explored the impact of the *miR‐126/DNMT1* axis on the proliferation and growth of leukaemia stem cell (LSC) lines, including MOLM13‐LSCs and KG‐1a‐LSCs. They firstly indicated a notable upregulation of *miR‐126* expression in both CD34+ cells and the aforementioned LSC lines. They observed that *miR‐126* silencing in MOLM13‐LSCs and KG‐1a‐LSCs impeded cellular proliferation while enhancing apoptosis. They further substantiated that *miR‐126* directly interacts with *DNMT1* and exerts negative regulatory control over its expression. Thereby, *miR‐126* enhances the proliferative capacity of LSCs by regulating *DNMT1* [159](Figure 4).

## 7 Therapeutic Perspective of LncRNAs/miRNA‐DNMT1 Axis: From Traditional Therapy to Novel Therapy

Recent empirical evidence indicated the role of the ncRNA/*DNMT1* axis in advancing malignant tumours. Consequently, this axis holds significant promise as a viable target for therapeutic intervention in managing human neoplastic conditions. Multiple ncRNA/*DNMT1* axis regulators have been formulated as potential interventions in cancer therapy. In the subsequent section, we delve into the significance of the ncRNA/*DNMT1* axis as a focal point for various remedies to combat malignancies in human beings.

### 7.1 LncRNAs/miRNA‐DNMT1 Axis Modulation via Herbal Medicine in Cancer Therapy

There has been a growing global acceptance of herbal medicines in recent years, leading pharmaceutical companies to actively explore them as valuable reservoirs for exploring novel drugs [160]. Empirical investigations have revealed that herbal medicine exhibits the potential to regulate various ncRNAs and the *DNMT1* axis, which are closely associated with cancer. Consequently, this modulation mechanism holds promise in impeding the onset and progression of cancer. The genus Vitex encompasses 250 shrubs and trees, distributed predominantly across the tropical and subtropical regions, while several species inhabit temperate zones. Traditionally, Vitex species have been historically employed to alleviate various health conditions, including premenstrual issues, migraines, malignancies, diarrhoea, respiratory infections, rheumatic pain, GI ailments, sprains and inflammatory responses. Casticin (3′, 5‐dihydroxy‐3, 4′, 6, 7 tetramethoxyflavone), a flavonoid compound possessing a molecular formula of C19H18O8 and a molecular weight of 374.34, holds significance in this regard. A commercially accessible variant of casticin (98% purity) derived from *V. trifolia* is readily obtainable in an analytically graded form. Casticin, a bioactive compound, has been extracted from different plant tissues within the Vitex genus, including the fruits and leaves of *V. trifolia* , aerial parts and seeds of *V. agnus‐castus* , and leaves of *V. negundo* . Recent studies demonstrated that casticin displays apoptosis and antiproliferation activity. This compound has shown effectiveness against numerous cancer cell lines through diverse molecular mechanisms [161]. CAS exhibited a selective decrease in the viability of HCC cells while having no discernible effect on L02 cells. Additionally, CAS demonstrated the ability to impede the stemness characteristics within HCC cells. CAS could suppress the activity and expression of *DNMT1* while simultaneously upregulating the levels of miR‐148a‐3p. Furthermore, the influence of CAS on stemness traits was nullified when *DNMT1* was stably overexpressed, whereas *miR‐148a‐3p* upregulation augmented the diminishing effect of CAS on stemness features. Further, *DNMT1* upregulation facilitated hypermethylation of the *miR‐148a‐3p* promoter, subsequently suppressing its expression. Additionally, *miR‐148a‐3p* effectively restrained DNMT1 expression by selectively binding to the 3′‐UTR of *DNMT1* mRNA. In the context of in vivo nude mouse xenograft experiments, agomir‐148a‐3p and CAS exhibited substantial efficacy in inhibiting tumour growth, surpassing the individual activities of either molecule. In this manner, CAS could impede stemness properties in HCC cells through its disruption of the mutual negative modulation between *miR‐148a‐3p* and *DNMT1* [162]. Importantly, the botanical remedy known as Rhizoma of Paris polyphyllin, a component of Traditional Chinese Medicine, has gained significant recognition among herbal healthcare professionals for its extensive use in treating various tumour types, such as those affecting the liver, urinary bladder and pancreas. Polyphyllin I (PPI), a steroidal saponin, has been extensively investigated as a prominent active constituent of Rhizoma of Paris. It has demonstrated noteworthy antitumor properties across various cancer types by impeding tumour cell proliferation, suppressing metastasis and eliciting cell cycle arrest and apoptosis via the mitochondrial pathway [163]. PPI exerted a substantial inhibitory effect on the proliferation and migration capabilities of CRPC cells while also inducing cell cycle arrest. Mechanistically, PPI led to a reduction in the expression of *HOTAIR*, *DNMT1* and *EZH2*. Intriguingly, *HOTAIR* silencing resulted in decreased protein expressions of *EZH2* and *DNMT1*. Conversely, the introduction of exogenous HOTAIR counteracted the inhibitory effects of PPI on *EZH2* and *DNMT1* protein expressions, as well as *EZH2* promoter activity and cell growth. Moreover, in vivo findings demonstrated that PPI triggers inhibition of tumour growth, *HOTAIR* and the protein expressions of *DNMT1* and *EZH2*. Therefore, PPI impedes the proliferation of CRPC cells by suppressing HOTAIR expression, subsequently leading to the repression of *DNMT1* and *EZH2* expressions. In this manner, the overall responses of PPI are influenced by the intricate interplay between *DNMT1*, *HOTAIR* and *EZH2*, characterised by their mutual regulation and reciprocal effects [164].

### 7.2 LncRNAs/miRNA‐DNMT1 Axis Modulation via Bioactive Molecules in Cancer Therapy

Curcumin, derived from the rhizome of the *Curcuma longa* plant and belonging to the polyphenolic class, has traditionally been utilised in medicinal practices as an agent with antioxidant and anti‐inflammatory properties [165]. However, the hydrophobic characteristics inherent to this phytochemical impose significant constraints on its ability to be effectively absorbed by cells and exert its biological effects. To surmount this challenge, a potentially efficacious strategy involves the incorporation of curcumin within dendrosome nanoparticles, which has recently been devised as dendrosomal nano‐curcumin (DNC) [166]. Chamani et al. explored the impact of DNC on the mir‐34 family member's expression in two HCC cell lines, Huh7 and HepG2. They demonstrated that DNC treatment induced upregulation of mir34a, mir34b and mir34c expression while concurrently downregulating the expression of DNMT1, *DNMT3A* and *DNMT3B* in both Huh7 and HepG2 cell lines. Also, the viability of Huh7 and HepG2 cells diminished by DNC administration, primarily by facilitating the reestablishment of *miR‐34 s* expression. So, DNC exerted its effect by downregulating DNMTs, thereby reactivating the epigenetically suppressed *miR‐34* family. In this manner, DNC could be a promising candidate for epigenetic therapy in HCC [167].

### 7.3 LncRNAs/miRNA‐DNMT1 Axis Modulation via Synthetic RNA Molecules in Cancer Therapy

Over the past 2 years, endeavours in synthetic biology have yielded innovative synthetic RNA constituents that can modulate gene expression within living organisms [168]. These advancements have laid the foundation for achieving scalable and customizable cellular functionality. The primary obstacles that need to be addressed in this nascent discipline involve elucidating strategies for effectively integrating computational and directed‐evolution techniques to enhance the intricacy of engineered RNA systems [169]. Additionally, there is a pressing need to explore avenues for the widespread application of these systems within mammalian contexts. PAS1‐30 nt‐RNA represents a chemically engineered PAS1 segment artificially created to incorporate enhancements in 2′‐O‐methylation and 5′‐cholesterol, specifically facilitating in vivo RNA transportation. In BC, *DNMT1* acts as a suppressor of PAS1 expression, and subsequent *DNMT1* silencing resulted in a noticeable increase in PAS1 levels. Additionally, protein PAS1 interacts with the RNA‐binding protein vigilin, preserving its overall stability. Furthermore, PAS1 facilitates the binding of H3K9me3 at the PH20 promoter through its interaction with SUV39H1, resulting in the repression of PH20. Importantly, in vivo and in vitro analysis revealed that PAS1 upregulation effectively impeded BC cell proliferation and metastasis. Combining decitabine with PAS1‐30 nt‐RNA significantly displays enhanced anti‐tumour effects, surpassing the efficacy observed with decitabine as a standalone treatment. The observed effectiveness of the combination is contingent not only upon the collaborative impacts of the DNMT inhibitor and PAS1‐30 nt‐RNA but also on the augmented expression of PAS1 instigated by the DNMT inhibitor. In this manner, in future BC treatment, a potential approach could involve the concurrent administration of decitabine and PAS1‐30 nt‐RNA, primarily targeting the modulation of *DNMT1*/*PAS1*/*PH20* interactions [170].

### 7.4 LncRNAs/miRNA‐DNMT1 Axis Modulation via miRNA Replacement in Cancer Therapy

MicroRNA molecules play a pivotal role in cancer progression and are progressively being implemented in clinical settings as targets and agents for therapeutic purposes [171]. A novel intervention strategy known as miRNA replacement has been recently devised, aiming to address the therapeutic potential of miRNAs. The rationale for advancing miRNA therapeutics is founded on the principle that rectifying these deficiencies in miRNAs through either antagonistic or restorative measures holds the potential to yield therapeutic advantages [172]. Therefore, we presented the most recent inquiries into the therapeutic approaches concerning the delivery of miRNAs. Specifically, Ding et al. examined the impact of miR‐200 family constituents and epigenetic alterations on preserving the mesenchymal/metastatic phenotype subsequent to EMT in HCC. They observed that mesenchymal cells following EMT exhibit significant upregulation of E‐box repressors *Zeb2* and *Zeb1*, alongside a simultaneous decrease in the expression of four members of the *miR‐200 family* (namely, *miR‐200a*, *miR‐200b*, *miR‐200c* and *miR‐429*). Their further experimentation revealed the methylation of multiple CpG sites present within the *E‐cadherin* promoter region in mesenchymal cells. They also showed that *miR‐200b* enforced expression in these cells led to a noteworthy enhancement in *E‐cadherin* levels and a concurrent decrease in cell migration in vitro. On the contrary, their in vivo investigations demonstrated the absence of notable alterations in metastatic capacity after *miR‐200b* overexpression. Their subsequent experimentation unveiled that the combined administration of a DNMT inhibitor and *miR‐200b* overexpression led to a considerable reduction in the invasive characteristics and complete elimination of metastatic potential in mesenchymal cells. Additionally, it was revealed that the specific application of short hairpin RNA to target *E‐cadherin* directly did not lead to the restoration of metastatic capability following DNMT silencing and re‐expression of *miR‐200b*. Furthermore, they disclosed that *E‐cadherin* restoration in primary mesenchymal cells proved insufficient in impeding metastatic potential. A practical approach to address liver cancer metastasis may involve a combined therapeutic strategy involving the modulation of miR‐200b expression and DNMT silencing without necessarily relying on *E‐cadherin* restoration [173]. Furthermore, Cai et al. examined the combined therapeutic impact of sorafenib and gold nanoparticles carrying anti‐*miR‐221* on HCC cell lines. Their investigation revealed that the administration of sorafenib in HepG2 and Huh7 cells triggered *miR‐221* signalling pathway activation, resulting in significant upregulation of *miR‐221* expression. They additionally validated the decrease in *p27* expression due to sorafenib treatment while observing a corresponding increase in *DNMT1* levels. They observed that increasing concentrations of AuNPs‐anti‐miR221 inhibited cell growth in both Huh7 and HepG2 cells. Moreover, the combined treatment of AuNPs‐anti‐miR221 and sorafenib led to a significant enhancement in cell growth inhibition. Additionally, they found that AuNPs‐anti‐miR221 exhibited a synergistic effect, further enhancing the inhibitory action of sorafenib. Their further experimentation disclosed that the administration of sorafenib in combination with AuNPs‐anti‐miR221 triggers elevated levels of p27 expression and reduced levels of DNMT1 expression. This signifies that AuNPs‐anti‐miR221 exhibits chemosensitizing properties when used in conjunction with sorafenib. Thereby, AuNPs‐anti‐miR‐221 could effectively augment the inhibitory impact of sorafenib on cell proliferation by deactivating the *miR‐221/p27/DNMT1* signalling pathway. Hence, it is plausible to consider AuNPs‐anti‐miR221 as a viable chemosensitizer in treating HCC when used with sorafenib [174]. Importantly, Indoleamine 2, 3‐dioxygenase (IDO) is an intracellular enzyme whose increased activity demonstrates a negative correlation with the presence of tumour‐infiltrating lymphocytes (TILs) in cases of oesophageal and endometrial cancers. Zhou et al. explored the impact of cancer‐secreted exosomal *miR‐142‐5p* on the immune status of cervical squamous cell carcinoma (CSCC). They initially demonstrated a positive association between elevated levels of *miR‐142‐5p* and indoleamine 2, 3‐dioxygenase (IDO) expression in lymphatic vessels associated with advanced CSCC. They observed that *miR‐142‐5p* is conveyed from CSCC‐secreted exosomes to lymphatic endothelial cells (LECs), leading to the depletion of CD8^+^T cells through the enhancement of lymphatic indoleamine 2, 3‐dioxygenase (IDO) expression. This effect was negated when an IDO inhibitor was administered. Their mechanistic analysis demonstrated that *miR‐142‐5p* directly inhibits the expression of lymphatic AT‐rich interactive domain‐containing protein 2 (*ARID2*). Furthermore, it hinders the recruitment of *DNMT1* to the interferon (*IFN)‐γ* promoter and amplifies the transcription of *IFN‐γ* by suppressing promoter methylation. Consequently, this cascade of events culminates in heightened IDO activity. They additionally observed a positive association between elevated levels of serum exosomal *miR‐142‐5p* and the advancement of CSCC, along with parallel increases in IDO activity. Therefore, CSCC cells release exosomes containing *miR‐142‐5p*, which subsequently promote IDO expression in LECs through the *ARID2‐DNMT1‐IFN‐γ* signalling pathway, resulting in the suppression and depletion of CD8^+^ T cells [175](Table 2).

**TABLE 2:** An overview of different compounds targeting non‐coding RNAs and their potential influence on DNMT1 activity.

Compound	Source	Cancer type	Target ncRNAs	Influence on DNMT1 activity	Ref.
Casticin	Herbal	Hepatocellular carcinoma	miR‐148a‐3p	Decrease	[162]
Polyphyllin I	Herbal	Castration‐resistant prostate cancer	LncRNA HOTAIR	Decrease	[164]
Dendrosomal Nano‐Curcumin	Modified herbal	Hepatocellular carcinoma	MiR‐34a/b/c	Decrease	[167]
Synthetic RNA (PAS1‐30 nt‐RNA)	Synthetic RNA	Breast cancer	LncRNA PHACTR2‐AS1	Decrease	[170]
miRNA Replacement (miR‐200b)	Synthetic miRNA	Hepatocellular carcinoma	miR‐200b	Decrease	[173]
AuNPs‐anti‐miR‐221	Synthetic anti‐miRNA	Hepatocellular carcinoma	miR‐221	Decrease	[174]
Exosome‐derived miR‐142‐5p	Exosomal miRNAs	Cervical squamous cell carcinoma	miR‐142‐5p	Decrease	[175]

## 8 Conclusion

Despite the notable progress made in diagnosis and treatment over recent decades, human cancer continues to pose a significant clinical obstacle owing to the lack of advancements in long‐term survival rates. Besides genetic change, disruption of epigenetic processes can also lead to altered gene function and malignant cellular transformation. Epigenetic enzymes such as *DNMT1* could lead to transcription repression by catalysing genomic DNA methylation and are usually aberrantly expressed in human tumours. Moreover, dysregulation of ncRNAs is linked to epigenetic reprogramming throughout tumour advancement, primarily attributable to their capacity to engage with DNMTs, notably *DNMT1*. In the current work, we noticed a reciprocal relationship between ncRNAs and *DNMT1*. Some miRNAs, including *miR‐185*, *miR‐139‐5p* and *miR‐377*, could directly target *DNMT1*, whereas others, such as *miR‐378*, *miR‐30b, miR‐34a*, *miR‐497* and *miR‐142* could be hypermethylated by *DNMT1* and downregulated. This dual regulatory mechanism further emphasises the complexity of miRNA‐*DNMT1* interactions and their relevance in cancer pathogenesis. Notably, the ncRNA‐*DNMT1* axis plays a critical role in mediating resistance to various chemotherapy agents, including cisplatin, doxorubicin and TMZ, by regulating the expression of essential miRNAs and promoting aberrant DNA methylation that impacts tumour cell sensitivity. In addition, the ncRNA‐*DNMT1* axis plays a crucial role in regulating CSCs activity, with multiple microRNAs, such as *miR‐34a* and *miR‐126*, modulating *DNMT1* expression to influence stemness characteristics and tumour progression across various cancers. Additionally, various therapeutic strategies, including herbal medicine, synthetic RNA molecules, DNC and miRNA replacement, have been implemented to modulate the ncRNA/*DNMT1* axis as part of cancer therapy approaches. However, one limitation of the current review article is that we mainly focused on two mechanisms by which lncRNAs regulate *DNMT1* function: first, by acting as molecular sponges for miRNAs, leading to increased *DNMT1* expression, and second, by functioning as scaffolds to recruit *DNMT1* to target miRNAs, resulting in their hypermethylation and suppression. However, lncRNAs can also operate through other approaches. For example, lncRNAs can interact with DNA and co‐transcriptionally form RNA–DNA hybrids, such as R‐loops, which are recognised by chromatin modifiers to either activate or inhibit target gene transcription, or by transcription factors. This mechanism, however, has not yet been studied in relation to *DNMT1*. Thus, one of the major limitations of the current work is that we did not cover all regulatory pathways related to lncRNAs in the regulation of *DNMT1*.

## 9 Future Research Perspective

Recent developments in gene editing technology have demonstrated promising approaches for precise and targeted DNA modification. Clustered Regularly Interspaced Short Palindromic Repeats (CRISPR)/Cas9, initially identified in *Escherichia coli* , provides a powerful tool for precise genome editing. By utilising base complementary pairing, the CRISPR/Cas9 system offers a highly specific DNA modification. Recently developed CRISPR/Cas9‐based tools, namely CRISPR interference (CRISPRi), employ a catalytically dead Cas9 (dCas9) protein complexed with a transcriptional effector and a single guide RNA (sgRNA). This variant of dCas9 is unable to trigger DNA cleavage, yet it maintains its capacity for sequence‐specific DNA binding. The binding of a dCas9/sgRNA complex to a target gene sequence modulates transcriptional activity [176]. Another variant of the CRISPR system, known as CRISPR activation (CRISPRa), can be utilised to enhance the expression of lncRNA genes. Recent studies have highlighted the potential of CRISPRa in activating DANCR, which in turn promotes chondrogenic differentiation and improves calvarial bone healing [177]. So, applying CRISPR/Cas9 technology could restore the expression of downregulated ncRNAs, leading to epigenetic reprogramming in various diseases, such as cancer. In this manner, CRISPR‐based targeted activation of ncRNAs such as miRNAs and lncRNAs may provide an alternative therapeutic approach for cancers.

## Author Contributions

**Seyed Mohsen Aghaei‐Zarch:** conceptualization (lead), supervision (lead), visualization (equal), writing – original draft (equal), writing – review and editing (equal). **Ali Esmaeili:** conceptualization (supporting), validation (supporting), visualization (equal), writing – original draft (equal), writing – review and editing (equal). **Saeid Bagheri‐Mohammadi:** conceptualization (equal), supervision (equal), validation (equal), writing – original draft (equal), writing – review and editing (equal).

## Ethics Statement

The authors have nothing to report.

## Conflicts of Interest

The authors declare no conflicts of interest.

## Data Availability

All the data generated is included within the manuscript.
